# Doubly zwitterionic, di-reduced, highly electron-rich, air-stable naphthalenediimides: redox-switchable islands of aromatic–antiaromatic states†
†Electronic supplementary information (ESI) available. CCDC 1822817–1822820. For ESI and crystallographic data in CIF or other electronic format see DOI: 10.1039/c9sc00962k


**DOI:** 10.1039/c9sc00962k

**Published:** 2019-05-21

**Authors:** Sharvan Kumar, Jyoti Shukla, Kalyanashis Mandal, Yogendra Kumar, Ravi Prakash, Panch Ram, Pritam Mukhopadhyay

**Affiliations:** a Supramolecular and Material Chemistry Lab , School of Physical Sciences , Jawaharlal Nehru University , New Delhi 110067 , India . Email: m_pritam@mail.jnu.ac.in; b School of Physical Sciences , Jawaharlal Nehru University , New Dehi 110067 , India

## Abstract

The di-reduced state of the naphthalene moiety and its congeners have long captivated chemists as it is elusive to stabilize these intrinsically reactive electron-rich π-systems and for their emergent multifaceted properties. Herein we report the synthesis and isolation of two-electron (2e^–^) reduced, highly electron-rich naphthalenediimides (NDIs). A doubly zwitterionic structure is observed for the first time in a naphthalene moiety and validated by single crystal X-ray crystallography and spectroscopic methods. The synthesis avoids hazardous reducing agents and offers an easy, high-yielding route to bench-stable di-reduced NDIs. Notably, we realized high negative first oxidation potentials of up to –0.730 V *vs.* Fc/Fc^+^ in these systems, which establish these systems to be one of the strongest ambient stable electron donors. The study also provides the first insights into the NMR spectra of the di-reduced systems revealing a large decrease in diatropicity of the naphthalene ring compared to its 2e^–^ oxidized form. The NICS, NICS-*XY* global ring current, gauge-including magnetically induced current (GIMIC) and AICD ring current density calculations revealed switching of the antiaromatic and aromatic states at the naphthalene and the imide rings, respectively, in the di-reduced system compared to the 2e^–^ oxidized form. Notably, the substituents at the phosphonium groups significantly tune the antiaromatic–aromatic states and donor ability, and bestow an array of colors to the di-reduced systems by virtue of intramolecular through-space communication with the NDI scaffold. Computational studies showed intramolecular noncovalent interactions to provide additional stability to these unprecedented doubly zwitterionic systems.

## Abstract 2

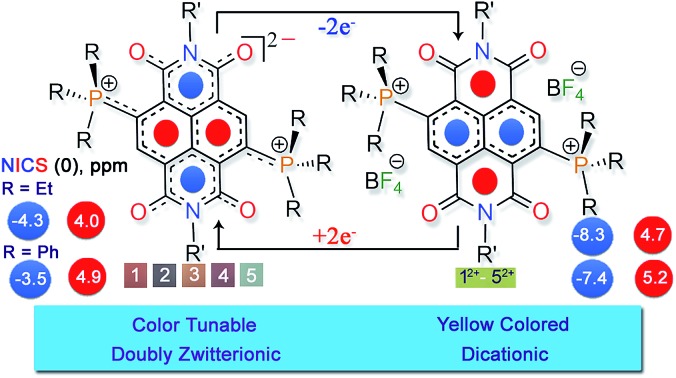
The synthesis and isolation of two-electron reduced naphthalenediimides is reported. A doubly zwitterionic structure is observed for the first time in a naphthalene moiety, which aids in its stabilization.

## Introduction

Insight into the stability of multi-electron accumulated states is crucial to develop super-reductants, conductors, and electrode materials in batteries, solar cells, *etc.*^1^–^4^ Also, realization of super-electron rich systems is of value as their excited states can be applied towards metal-free organic transformations.^5^ However, π-conjugated systems exhibiting high negative oxidation potentials are intrinsically unstable under ambient conditions.^6^ There is also great interest to discern the effects of electron accumulation on the ring currents of these π-systems.^7^ As seen recently, intriguing electronic scenarios evolve if aromatic and antiaromatic states co-exist in π-systems.^8^ Understanding and control over these states may lead to realization of a rare class of bench-stable electron-rich π-systems. Presently, rational design concepts are lacking that can facilitate multi-electron injection, restrict their high reactivity and address their isolation challenges.

**Scheme 1:**
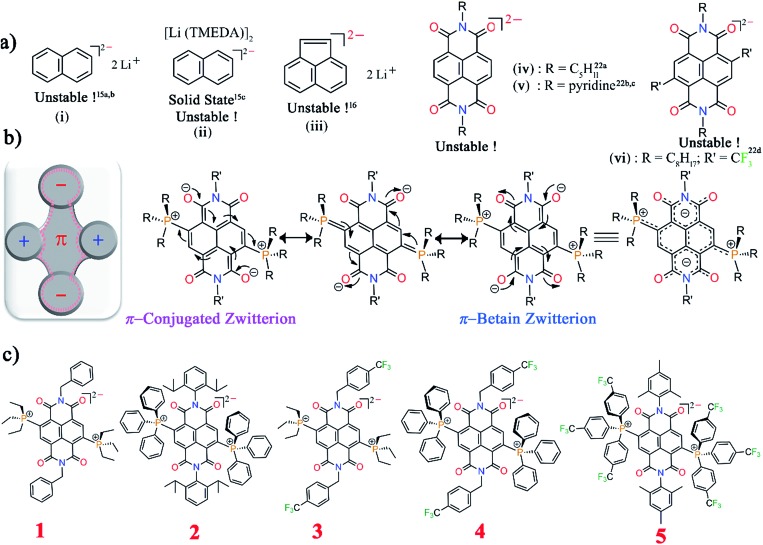
(a) Naphthalene-based reported di-reduced species. (b) Pictorial and molecular representation of π-conjugated and betaine type di-zwitterions and (c) molecular structures of di-reduced naphthalenediimides. TMEDA = tetramethylethylenediamine.

Isolation of a two-electron reduced naphthalene dianion and its π-extended systems is a challenge being pursued over last several decades. In 1965, a naphthalene dianion was realized in the solution state9*a* and later isolated in the solid state,9*b* while a π-extended acenaphthalene dianion^10^ was realized in solution (Scheme 1a). All these dianions are extremely reactive and are handled under inert conditions. Subsequently, arylenediimide π-scaffolds^11^ like naphthalenediimide (NDI) and perylenediimide (PDI) attracted huge interest due to their ability to accept two-electrons (2e^–^).^12^–^14^ While the 1e^–^ reduced NDI radical ion has been isolated,^15^,^16^ isolation of the highly electron-rich di-reduced NDI remains elusive (Scheme 1a). In an elegant synthetic development, the 2e^–^ reduced PDI dianion has recently been isolated.^14^ PDI with its π-extended structure can aid significantly in charge stabilization *vis-à-vis* the smaller NDI congener. Although through-space charge delocalization has been elegantly formulated in NDI cyclophanes, ambient stabilization of 2e^–^ reduced individual NDIs has not been possible.^17^ Further, there have been considerable efforts to apply the photo-excited states of these electron-rich systems.^18^,^19^ Ground-state ambient stabilization and their stoichiometric usage is anticipated to open-up new vistas in controlling electron transfer reactions. We envisaged to explore the effect of 2e^–^ uptake and release as well as the tunability of the aromatic naphthalene π-backbone and the nonaromatic imide rings. Thus, redox-assisted inter-conversion between the aromatic and nonaromatic states can be envisioned.

Herein we report the first synthesis and isolation of highly electron-rich di-reduced NDIs. The doubly zwitterionic structure employed in the naphthalene scaffold affords an appealing mode of charge distribution and ambient stabilization (Scheme 1b). A facile eco-friendly synthetic protocol was rationalized from the electrochemical data and leads to strong electron donors (**1–5**) with the first oxidation potential ranging from –0.724 to –0.263 V *vs.* Fc/Fc^+^ (Scheme 1c). The single crystal X-ray structures for the di-reduced systems were compared with the corresponding 2e^–^ oxidized dications.

Theoretical calculations showed that additional electrons are delocalized over the NDI unit and the carbonyl O atoms embrace bulk of the negative charge. On the other hand, the phosphonium groups contain majority of the positive charge. Such an arrangement of the opposite charges at two different sites within the molecule endows it with di-zwitterionic character. From the resonating structures, a hybrid of a π-conjugated zwitterion and a π-betaine zwitterion can be obtained as shown in Scheme 1b.

## Results and discussion

### Synthesis

**Scheme 2:**
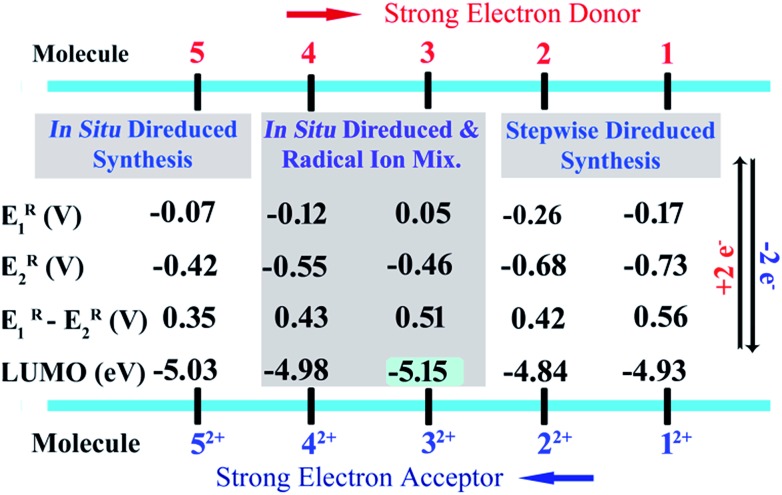
Reduction potentials against Fc/Fc^+^ in DCM. The LUMO energies were calculated as *E*_LUMO_ = [–5.1(Fc) – *E*R1] eV against vacuum on the basis of CV experiments.

Herein we report the first synthetic protocol towards the isolation of ambient stable NDI-based di-reduced compounds (**1–5**). We observed that second reduction potential of the dicationic NDI (2e^–^ oxidized form) plays a key role during the synthesis of di-reduced compounds. EWGs at the axial- and the core-positions of the NDI led to a significant contrast between the first and second reduction potentials, which significantly favoured the one-step synthesis of **3–5**. The reduction potentials and the corresponding LUMO levels of the parent dicationic compounds are listed in Scheme 2.

**Scheme 3:**
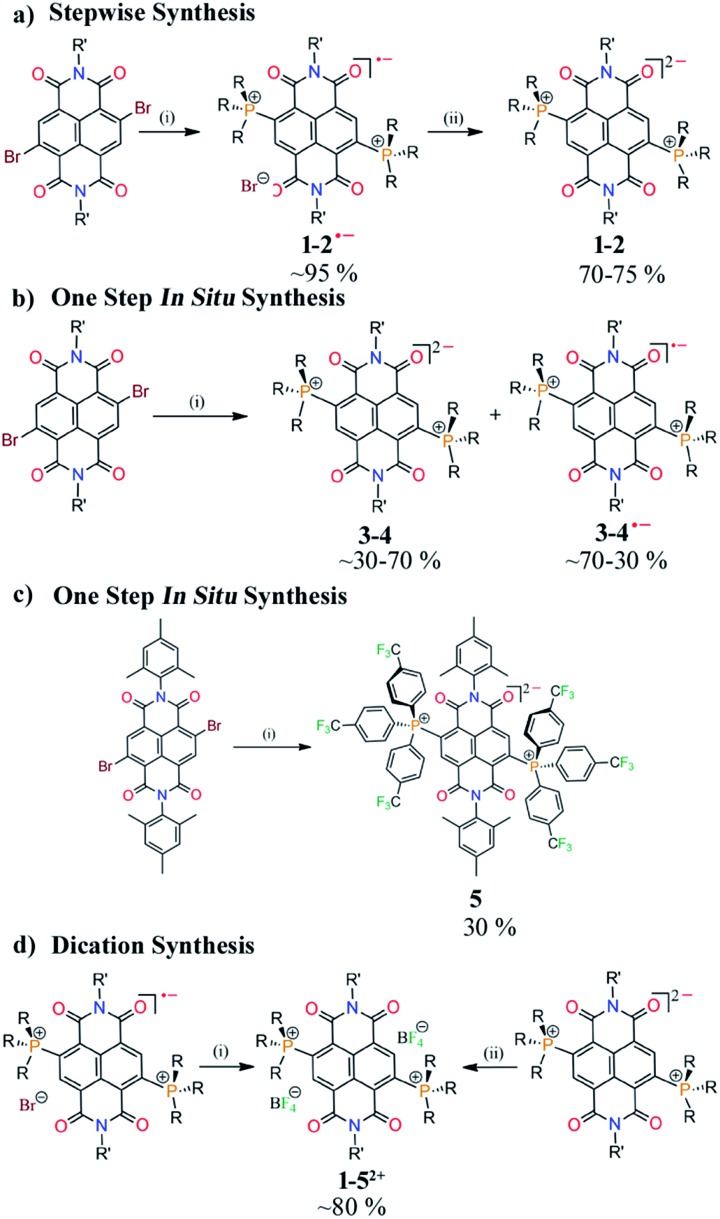
Synthesis of di-reduced NDIs. Reagents and conditions: (i) PR_3_, Et_3_N, 100 °C, 30 min. and (ii) Na_2_S in MeOH, MeCN, RT, 30 min. (a) Stepwise synthesis. (b) *In situ* synthesis of radical and di-reduced species. (c) *In situ* synthesis of di-reduced **5** and (d) synthesis of dications from radical ions as well as di-reduced compounds. Reagents and conditions: (i) and (ii) NOBF_4_, RT, 15 min.

We also realized a direct one-pot synthesis of **3** and **4** along with the corresponding radical ions when NDIs comprised of electron withdrawing groups (EWGs) at the axial positions (Scheme 3b). UV-vis absorption spectroscopy revealed the formation of di-reduced NDIs along with the radical ions (ESI Fig. S1†). The exclusive formation of **5** was observed when phosphenes were substituted with *p*-CF_3_Ph EWGs (Scheme 3c). Finally, we synthesized the corresponding dicationic compounds *via* one-electron oxidation of the radical ion or two-electron oxidation of the di-reduced compounds (Scheme 3d).

Importantly, **3^2+^** and **5^2+^** revealed exceptionally low LUMO levels of –5.15 and –5.03 eV, respectively. Interestingly, the LUMO of **3^2+^** is 0.25 eV lower than that of the ultra-electron deficient NDI dication,^16^ and establishes itself as the lowest LUMO arylenediimide to be reported to date. Thus, **3^2+^** is one of the strongest electron acceptors to be isolated.^14^,16a,^20^,^21^

Earlier we demonstrated the plausible mechanism for the *in situ* synthesis of diphosphonium substituted NDI radical ions.16*b* We anticipated that due to the contrast between the first and second reduction potentials in the dicationic systems embracing EWGs in the axial or core-positions, the radical ions would be able to accept an electron from the triethylamine to form *in situ* direduced compounds. To confirm this possibility, we performed a reaction of **1^2+^** and **4^2+^** with triethylamine at 100 °C. We observed gradual conversion of **4^2+^** to **4**, while **1^2+^** only formed the corresponding radical ion (ESI Fig. S2†). This confirmed that triethylamine can further reduce the radical ion and form the direduced species if the dication is substituted with EWGs. On the basis of our earlier findings and this result, we proposed the probable mechanism for the formation of the direduced compounds (ESI Scheme S1†).

### NMR spectroscopy

**Fig. 1:**
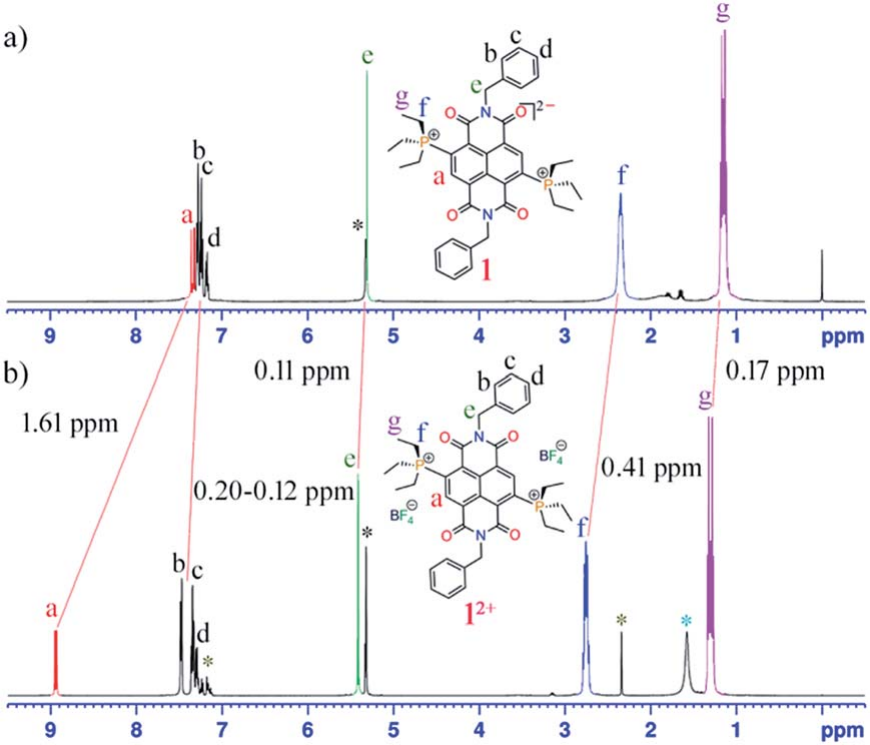
^1^H NMR spectra of (a) **1** and (b) **1^2+^** in DCM at room temperature. The large upfield chemical shifts in **1** compared to **1^2+^** are shown by red lines. (*) denotes solvent residual peaks of toluene (*, from crystallization), DCM (*) and water (*).

In the presence of an external magnetic field, aromatic systems sustain a diatropic ring current while antiaromatic systems sustain a paratropic ring current.^22^ The ^1^H NMR spectra of **1** shows a significant upfield chemical shift of all the protons compared to those of **1^2+^** (Fig. 1). In the case of **1**, the proton signal H_a_ corresponding to the proton of the naphthalene unit appears at 7.33 ppm (*J* = 17 Hz), which is shifted upfield by 1.61 ppm compared to the same protons of **1^2+^** that appear at 8.94 ppm (*J* = 11 Hz). This signifies a significant contribution of the paratropic ring current in the naphthalene ring and decrease in diatropicity. This upfield ^1^H NMR chemical shift is observable even for the protons not directly attached to NDI scaffold. For example, the protons H_f_ and H_g_ of the ethyl groups linked to the phosphonium groups in **1** appear at 2.34 and 1.13 ppm, respectively, while in **1^2+^** they appear at 2.75 and 1.30 ppm, respectively. The axial group protons, H_e_ (5.30 ppm) and H_b–d_ (7.28–7.15 ppm), of **1** also show measurable upfield chemical shifts compared to the same protons in **1^2+^** appearing at 5.41 and 7.48–7.27 ppm, respectively.

Such an upfield chemical shift in the ^1^H NMR spectra was also observed for **2–5**. The phenylene/naphthalene protons in **2** (ESI Fig. S25†), **4** (ESI Fig. S36†) and **5** (ESI Fig. S42†) appear from 7.79, 7.78 and 7.92 ppm, which is significantly shifted upfield compared to **2^2+^** (ESI Fig. S28†), **4^2+^** (ESI Fig. S39†) and **5^2+^** (ESI Fig. S44†), in which these protons begin from 8.42, 8.35 and 8.45 ppm, respectively. In **2**, the CH protons of the phenyl diisopropyl axial groups are 0.31 ppm shifted upfield compared to the CH protons of **2^2+^**. Likewise, in **5** the methyl protons of the axial mesityl groups displayed 0.11–0.07 ppm upfield shifts compared to **5^2+^**. In **3** (ESI Fig. S31†) with a triethyl phosphonium group at the NDI-core, the phenylene/naphthalene peaks start from 7.50 ppm, which is 1.39 ppm upfield shifted compared to **3^2+^** (ESI Fig. S34†), which appears at 8.89 ppm. The CH_2_ and CH_3_ protons of the phosphonium groups of **3** appear upfield shifted to 2.35 and 1.15 ppm respectively, compared to 2.75 and 1.31 ppm, respectively, in **3^2+^**.

The ^31^P NMR spectra of these di-reduced compounds also exhibited significant upfield chemical shifts of up to 11 ppm. Compounds **1**, **2**, **3**, **4** and **5** showed ^31^P peaks at 32.90, 25.45, 33.25, 25.87 and 24.89, respectively. In the corresponding **1^2+^**, **2^2+^**, **3^2+^**, **4^2+^** and **5^2+^** this peak appears at 44.07, 31.09, 43.53, 31.12 and 30.45 ppm, respectively. The ^13^C NMR of the di-reduced compounds showed measurable upfield chemical shifts in the ^13^C signals compared to the dications. These significant upfield ^1^H, ^31^P and ^13^C NMR chemical shifts suggest efficient delocalization of the two additional electrons in the di-reduced systems.

### FT-IR spectroscopy

The stretching frequencies of the imide carbonyl bonds of **1–5** are shifted by ∼50–120 cm^–1^ to lower frequencies compared to the dicationic compounds (ESI Fig. S11†). The imide carbonyl asymmetric and symmetric stretching frequencies for **1** were found to be 1648 cm^–1^ and 1617 cm^–1^, respectively, while for **1^2+^** they were 1715 and 1667 cm^–1^, respectively. Likewise, for **2** the respective frequencies were 1630 and 1558 cm^–1^, respectively, compared to 1716 and 1664 cm^–1^ for **2^2+^**. Compound **3^2+^** showed the corresponding frequencies of 1714 and 1664 cm^–1^ while for **3** it was observed to be 1619 and 1556 cm^–1^, respectively. The asymmetric and symmetric frequencies for **4^2+^** and **5^2+^** were found to be 1716 and 1658 cm^–1^ and 1720 and 1660 cm^–1^, respectively. They were found to be 1619 and 1558 cm^–1^ and 1634 and 1555 cm^–1^ for **4** and **5**, respectively.

### X-ray crystallography

To have the first insight into the structural characteristics of the di-reduced compounds, we sought to crystallize **1** and **2**. X-ray suitable single crystals of **1** and **2** were grown from DCM : toluene (2 : 1) solution.

Further, single crystals of **1^2+^** and **2^2+^** were grown from DCM: toluene (2 : 1) solution at room temperature and the structural parameters compared with **1** and **2**.^23^

#### Crystallographic studies of **1** and **1^2+^**

**Fig. 2:**
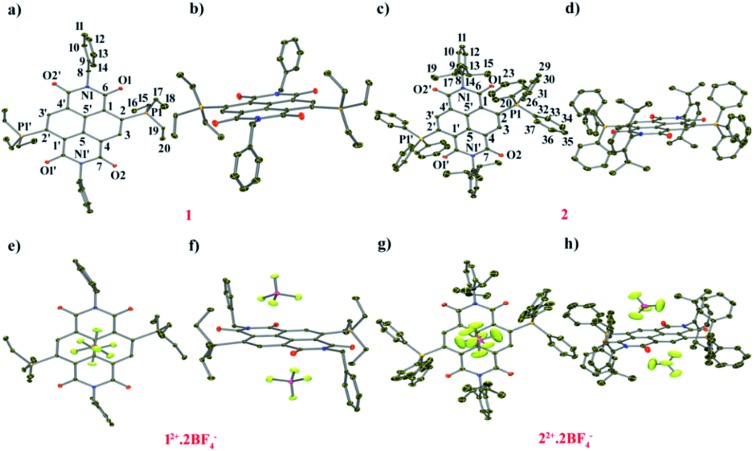
ORTEP representations of: [**1**] (a) top and (b) side view. Selected bond lengths (Å): C1–C2 1.456(4), C2–C3 1.371(4), C3–C4 1.423(4), C1–C5′ 1.405(4), C4–C5 1.409(4), C5–C5′ 1.437(4), C6–O1 1.252(4), C7–O2 1.244(4), N1–C6 1.394(4), N1′–C7 1.406(4), C2–P1 1.795(4), P1···O1, P1′···O1′ 2.806 and 2.818. ORTEP representations of [**2**]: (c) top and (d) side views. Selected bond lengths (Å): C1–C2 1.444(4), C2–C3 1.376(3), C3–C4 1.421(4), C1–C5′ 1.403(4), C4–C5 1.412(4), C5–C5′ 1.437(3), C6–O1 1.244(4), C7–O2 1.237(5), N1–C6 1.390(4), N1′–C7 1.415(5), C2–P1 1.779(3), P1···O1 2.658. ORTEP representations of (**1^2+^**)·2BF_4_^–^: (e) top and (f) side views. Selected bond lengths (Å): C1–C2 1.396(2), C2–C3 1.413(2), C3–C4 1.373(2), C1–C5′ 1.407(2), C4–C5 1.410(2), C5–C5′ 1.410(2), C6–O1 1.212(2), C7–O2 1.219(2), N1–C6 1.392(2), N1′–C7 1.386(1), C2–P1 1.827(1), P1···O1 2.811, B···Ct 3.524, F1···C1–5 3.113–3.451. (g) Top view and (h) side view of crystal structure of (**2^2+^**)·2BF_4_^–^. Selected bond lengths (Å): C1–C2 1.394(4), C2–C3 1.404(4), C3–C4 1.376(4), C1–C5′ 1.412(4), C4–C5 1.394(4), C5–C5′ 1.416(4), C6–O1 1.213(4), C7–O2 1.208(4), N1–C6 1.388(4), N1′–C7 1.404(4), C2–P1 1.824(4), P1···O1 2.738, B···Ct 3.565, F1···C1–5 2.968–4.010. H atoms have been removed for clarity. Thermal ellipsoids are shown at 50% probability.

**1** crystallizes in the monoclinic space group “*C*2” with one molecule per asymmetric unit (Fig. 2a and b). We observed significant increment in the C=O bond length of **1** compared to **1^2+^** and the longest C=O bond was found to be 1.252 Å, suggesting its partial double bond character. In addition, bond lengthening in the annular C5–C5′ and the transverse bonds of the naphthalene moiety along with shortening in the P1–C2, C2–C3, C1–C6 and C4–C7 bonds was observed indicating delocalization of the two additional electrons over the NDI scaffold (ESI Table S1†). The shortest intramolecular P···O non-bonded distance was found to be 2.806 Å, which is slightly shorter than that of **1^2+^** (*vide infra*).^24^ The bond lengthening of the C=O groups and shortening of the P···O distance suggest significant polarization of the positive and negative charges over the P atom of the phosphonium group and the O atom of the imide groups, respectively. A π-conjugated betaine-type di-zwitterionic structure is therefore pertinent from the crystallographic insight.

**1^2+^** crystallizes in the triclinic space group “*P*1[combining macron]” with a half molecule per asymmetric unit (Fig. 2e and f). The imide C=O adjacent to the phosphonium group shows a bond distance of 1.212 Å, while the C=O bond situated remote to the phosphonium group had a distance of 1.219 Å. The intramolecular P···O non-bonded distance was found to be 2.811 Å. The BF_4_^–^ counter anions show strong anion–π interaction with a small shift towards the imide region of the NDI scaffold. The distance between the F atom of anion and the C atoms of the naphthalene moiety is in the range of 3.113–3.451 Å and the distance from the centroid of naphthalene (Ct) to the B atom of the anion was found to be 3.524 Å.

#### Crystallographic studies of **2** and **2^2+^**

Compound **2** crystallizes in the triclinic space group “*P*1[combining macron]” with a half molecule per asymmetric unit (Fig. 2c and d). Furthermore, the longer C=O bond length of 1.224 Å in comparison to **2^2+^** (1.213 Å) corroborates partial double bond character. The C=O group remotely placed with respect to the phosphonium group also showed bond elongation (1.245 Å) in comparison to **2^2+^** (1.208 Å) (ESI Table S2†). The elongation of the annular C5–C5′, transverse (C1–C2, C3–C4 and C4–C5) and imide bonds (C6–N1 and C7′–N1) and shortening in the C2–C3, C1–C6 and C4–C7 bonds in **2** compared to **2^2+^** show that the two additional electrons are delocalized over the NDI scaffold. The distance between P atoms of the phosphonium groups to the adjacent O atoms of the imide groups was found to be 2.698 Å, which is 0.040 Å smaller than that of **2^2+^**.

Compound **2^2+^** crystallizes in the monoclinic space group “*P*2_1_/*n*” with a half molecule per asymmetric unit (Fig. 2g and h). The imide C=O adjacent to the phosphonium group shows a bond length of 1.213 Å, slightly longer than the C=O bond (1.208 Å) situated remote to the phosphonium group. The distance between P···O was found to be 2.738 Å, which is 0.073 Å shorter than that in **1^2+^**, corroborating a stronger P···O donor–acceptor type interaction when the phosphonium groups are substituted with phenyl rings. Moreover, both the BF_4_^–^ anions form anion–π interactions and are situated exactly on the top and bottom of the naphthalene ring with a distance of 2.968–4.010 Å between the naphthalene carbons and the F atom of BF_4_^–^ anions (ESI Table S2†).

### Electrochemistry

**Fig. 3:**
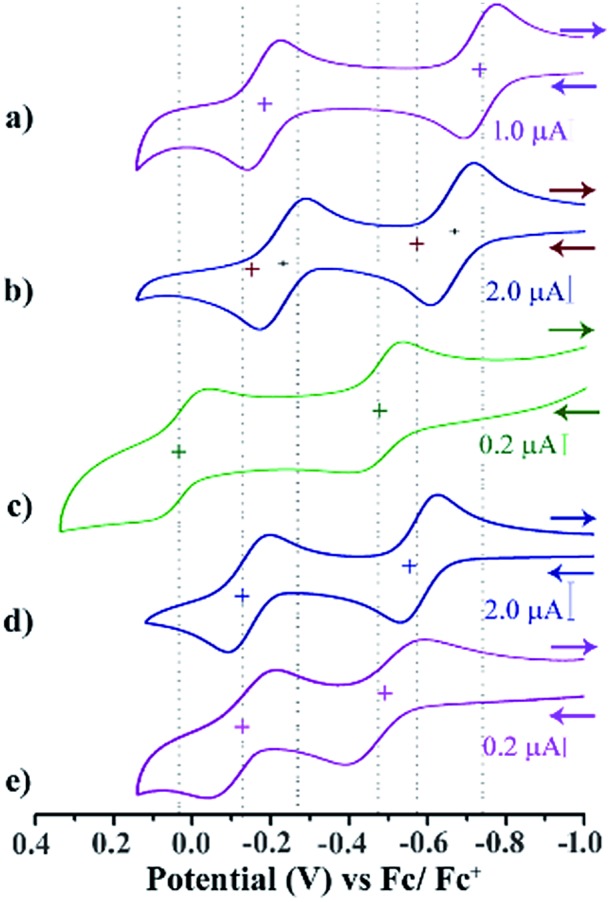
CV and DPV of di-reduced molecules (a) **1**, (b) **2**, (c) **3**, (d) **4**, (e) and **5**. Conditions: 5 × 10^–4^ M in DCM; reference electrode, Ag/AgCl; working and auxiliary electrodes, Pt with 0.1 M Bu_4_NPF_6_ and (Fc/Fc^+^); 298 K; scan rate, 200 mV s^–1^.

Cyclic voltammetry (CV) and differential pulse voltammetry (DPV) experiments were performed for the di-reduced and dicationic forms in DCM (ESI Fig. S3 and S4†). The CV and DPV studies of **1–5** exhibited two reversible oxidation peaks corresponding to the sequential two-step ET process (Fig. 3). Notably, the first oxidation potentials of **1** and **3** are shifted to more negative values than that of the second reduction potentials of the corresponding dicationic compounds (Table 1).

**Table 1:** Oxidation potentials of **1–5** determined by CV studies against Fc/Fc^+^ in DCM. HOMO level energies and UV-vis absorption spectroscopy^*a*^

Mol.	Potential (V) *vs.* Fc/Fc^+^			Exp. *λ*absmax [nm] (*ε*, L mol^–1^ cm^–1^)
	*E* OX 1 (Δ*E*_p_)	*E* OX 2 (Δ*E*_p_)	HOMO	
**1**	–0.73 (0.08)	–0.18 (0.08)	–4.36	572 (21 200)
**2**	–0.67 (0.11)	–0.23 (0.11)	–4.42	593 (21 600)
**3**	–0.47 (0.11)	0.06 (0.11)	–4.62	575 (18 100)
**4**	–0.55 (0.09)	–0.12 (0.09)	–4.54	584 (31 800)
**5**	–0.49 (0.18)	–0.12 (0.18)	–4.61	591 (33 900)

^*a*^Δ*E*_p_ is the potential difference between the anodic and cathodic peaks. HOMO energies were calculated as *E*_HOMO_ = [–5.1 (Fc) – *E*OX1] eV against vacuum on the basis of CV experiments.

The most negatively shifted oxidation potentials at –0.724 and –0.186 V *vs.* Fc/Fc^+^ was observed for **1** corresponding to the 1st and 2nd oxidation potential, respectively. The HOMO was determined to be –4.376 eV from the CV experiment. Compound **2** showed oxidation potentials at –0.573 and –0.140 V corresponding to a HOMO of –4.527 eV. On the other hand, **3**, **4** and **5** exhibited the 1st and 2nd oxidation potentials at –0.474 and 0.060 V, –0.554 and –0.122 V, and –0.263 and 0.095 V, respectively. The corresponding HOMO levels for **3–5** were found to be –4.626, –4.546 and –4.837 eV, respectively. Therefore, **1** is the strongest electron donor followed by **2**, **4**, **3** and **5**. Notably, **1** is one of the strongest ambient stable electron donors to be isolated to date.^25^ Notably, in this class of electron donors the HOMO level can be modulated by ∼0.5 eV by altering the substituents at the axial and the core positions of the NDI scaffold.

CV and DPV studies of **1^2+^–5^2+^** demonstrate two quasi-reversible reduction peaks corresponding to sequential two-step electron transfer processes. The reduction potentials of compounds **1^2+^–5^2+^** clearly showed the effect of the EWGs at the axial- and the core-substituents (Scheme 2). On comparison of the reduction potential data, it is clear that EWG substituents at the NDI-axial groups play a major role in decreasing both the first and second reduction potentials, while EWGs at the NDI-core predominantly affect the second reduction potential and significantly reduces the *E*R1 – *E*R2 potential gap. Moreover, the plot of the square root of the scan rate with respect to the current peak of both the redox waves suggested its thermodynamic stability and reversibility (ESI Fig. S5†).

### UV-vis spectroscopy

**Fig. 4:**
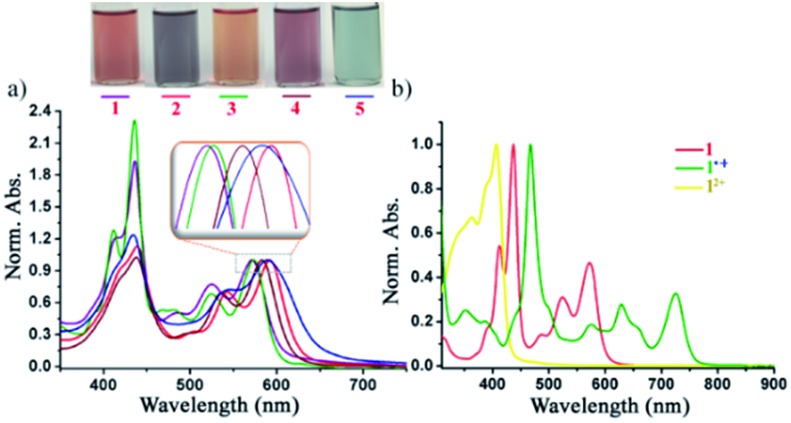
(a) Normalized UV-vis-NIR spectra of **1–5** in DCM. (b) Spectra showing the three-state spectra of **1**, **1˙^+^** and **1^2+^**.

The UV-vis absorption spectra of the di-reduced compounds in DCM exhibit characteristic spectroscopic features of the NDI dianion. All di-reduced compounds exhibited intense absorption bands in the 412–592 nm regions with the absorption coefficient (*ε*) reaching 3.3 × 10^4^ L mol^–1^ cm^–1^ (Fig. 4a and Table 1). Compound **1** absorbs at 412, 437, 523 and 571 nm, while **2** shows red-shifted absorption bands at 418, 440, 541 and 592 nm. The *λ*_max_ of **2** undergoes a 21 nm red-shift compared to **1** suggesting the role of the axial and core substituents in substantially influencing the frontier molecular orbital energies. A similar trend is observed on going from **3–5**. Fig. 4b shows the characteristics absorption bands of the other two oxidized states of **1**, *i.e.* the radical ion **1˙^+^** and the dication **1^2+^**.

Moreover, we could succeed in tuning the colour of the di-reduced compounds by changing the axial and core functional groups of the NDI (Fig. 4a). Compound **1** which has triethylphosphonium group at the NDI core and benzyl groups at the axial positions gives brownish-red colour, while on replacement of axial benzyl with *p*-CF_3_ benzyl in **3** the colour changes to orange-red.

On the other hand, **2** having phenyl phosphonium groups at the core with diisopropylphenyl as the axial substituents revealed a purple-blue colour, which can be further modulated to light purple colour in **4** and light blue in **5**. Next, we followed the gradual formation of **4** from the radical ion **4˙^+^** in the presence of a mild reducing agent like tetrabutyl ammonium cyanide in DMF by UV-vis-NIR absorption spectroscopy (ESI Fig. S6†). The complete conversion to the di-reduced compound was confirmed by the complete disappearance of the characteristic absorption bands of the radical ion **4˙^+^** at 469, 630 and 724 nm and the appearance of the characteristic bands for **4** at 436, 499, 536 and 584 nm.

### Stability in the solution state

The solution stability of a representative compound **4** was determined in different solvents through UV-vis-NIR absorption spectroscopy. The *λ*_max_ absorption band was followed to calculate the half-life time (*t*_1/2_). In toluene : DCM (97 : 3 v/v), the *t*_1/2_ was found to be 6.1 months, which is the highest stability amongst any reported di-reduced NDI molecules (ESI Fig. S7†). In DCM the *t*_1/2_ was found to be 51.4 days. On the other hand, in polar aprotic solvents like THF, MeCN, DMF and DMSO the *t*_1/2_ ranges between ∼3 and 4 days. These results confirmed that in nonpolar solvents the di-reduced compound has significantly higher stability. It is to be noted that all the experiments were done under ambient conditions. The trace impurities including water present in polar solvents possibly reduce the stability of the direduced compounds. We also examined stability of **4** in the solid-state by exposing it to open air for several months and found that there are no significant changes in the absorption spectrum albeit formation of a negligible amount of the radical ion.

### NICS computations

Next, we performed nucleus independent chemical shift (NICS) calculations. The negative NICS values indicate a diatropic ring current, while positive NICS values indicate a paratropic ring current.^26^ NICS (0) and NICS (1) values result from the calculated magnetic shielding tensor for a “dummy” atom located in the center of the ring and 1 Å above/below the plane of the ring system under examination, respectively. The NICS values of **1** and **2** are compared with the corresponding NICS values of **1^2+^** and **2^2+^**, respectively (Table 2). NICS (0) and NICS (1) values of –8.3 and –10.3, and –7.4 and –9.6 ppm for the naphthalene ring of **1^2+^** and **2^2+^**, respectively, indicate that the aromaticity is intact.

**Table 2:** NICS (0) and NICS (1) parameters for dication and di-reduced compounds

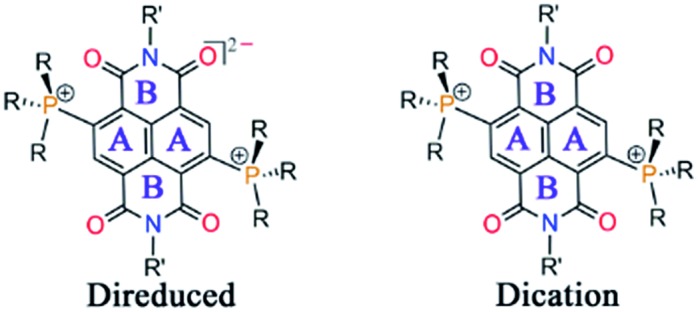								
	NICS (0), ppm				NICS (1), ppm			
	**1**	**2**	**1^2+^**	**2^2+^**	**1**	**2**	**1^2+^**	**2^2+^**
A	4.0	4.9	–8.3	–7.4	0.8	1.6	–10.3	–9.3
B	–4.3	–3.5	4.7	5.2	–5.9	–5.4	0.3	0.7

However, the positive NICS (0) values of 4.0 ppm and 4.9 ppm for the naphthalene ring in the case of the di-reduced systems **1** and **2**, respectively, indicate mild antiaromatic nature. On the other hand, the NICS (1) values for the same was found to be 0.8 and 1.6 ppm respectively, which indicates the nonaromatic nature of the naphthalene moiety.

In contrast, the imide regions of **1^2+^** and **2^2+^** comprising of the six-membered rings shows positive NICS (0) values of 4.7 and 5.2 ppm, respectively, indicating antiaromaticity. The NICS (1) values for the same were found to be 0.3 and 0.7 ppm, respectively, indicating nonaromatic nature. However in **1** and **2**, the NICS (0) turns negative with values of –4.3 and –3.5 ppm, indicating its weakly aromatic nature, while NICS (1) turns negative with with values of –5.9 and –5.4, suggesting its aromatic nature. These findings indicate that in the di-reduced compounds, the naphthalene moiety nurtures antiaromatic/nonaromatic states and the imide rings provide the aromatic/nonaromatic state. Thus, a 2e^–^ redox reaction can switch the antiaromatic–aromatic sites in these electron-rich and electron-deficient systems.

### NICS-*XY* scan

**Fig. 5:**
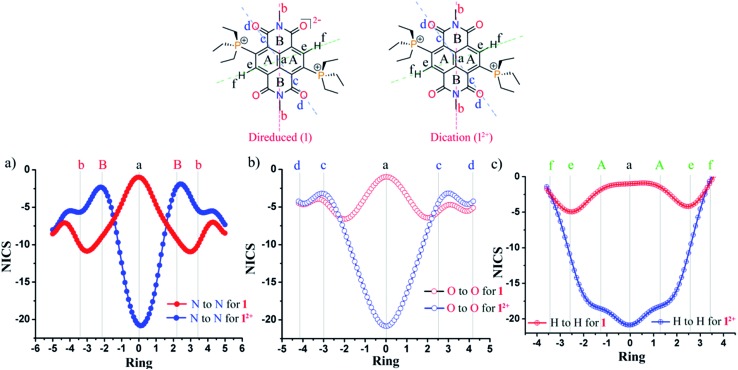
NICS-*XY* scans of **1^2+^** and **1**. (a) Along imide N to imide N (solid; red = **1**; blue = **1^2+^**), (b) imide O to imide O (hollow; red = **1**; blue = **1^2+^**) and (c) along the diagonal naphthalene hydrogen; H to H (red = **1**; blue = **1^2+^**).

**Fig. 6:**
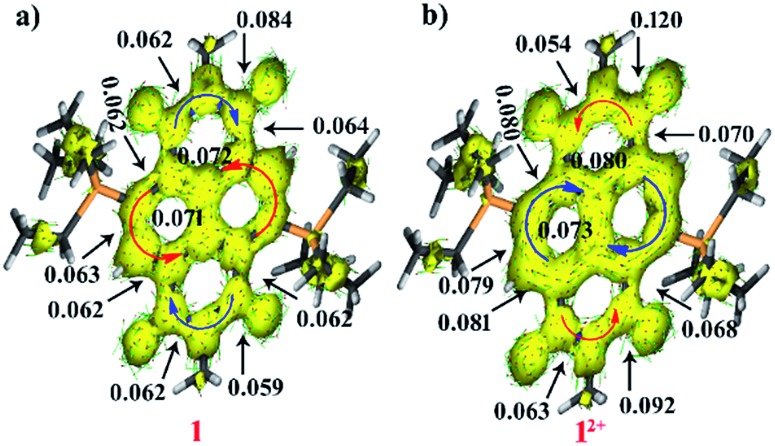
AICD isosurface plots of (a) **1**, and (b) **1^2+^**. The induced ring current vectors are plotted on the AICD isosurface to denote the diatropic and paratropic ring currents. The AICD plots are plotted at an isosurface value of 0.05.

To comprehend the global ring current, we performed the NICS-*XY* scans for the di-reduced systems and compared with the corresponding dications.^27^ We performed NICS-*XY* scan for **1** and **1^2+^** in different directions keeping NICS probes (dummy atoms) at 1.7 Å above the molecular plane under examination at 0.1 Å intervals and scanned along the *X*-axis (Fig. 5). The NICS-*XY* scan of 1 along the H′s of the naphthalene ring shows a NICSπ,*zz* value of –1.0 ppm at the centre, which indicates the nonaromatic nature of the naphthalene ring. It also shows a value of –4.9 ppm at the carbon centre bearing the H atom of naphthalene indicating a local diatropic current at these carbon centres (Fig. 5c). Further, the NICS-*XY* scan along the imide N atoms shows NICSπ,*zz* values of –1.0 ppm indicating the nonaromatic nature at the centre of the naphthalene ring. This imide N to imide N NICS-*XY* scan also shows a value of –10.8 ppm at the centre of the imide rings indicating significant diatropic current and the aromatic nature of the imide rings (Fig. 6a). The O to O NICS-*XY* scan shows a value of –6.6 ppm at the periphery of the imide ring also suggesting the aromatic nature of the imide rings (Fig. 5b). The NICS-*XY* scan of the dication molecule **1^2+^** along one of the Hs of the naphthalene ring to another H of the same shows NICSπ,*zz* values of –20.8 ppm indicating a diatropic current within the boundaries, suggesting aromatic nature. The imide N to imide N and imide O to imide O NICS-*XY* scans also show NICSπ,*zz* values of –20.8 ppm indicating diatropic current at the centre and a value of –2.2 ppm at the imide ring which indicates the weakly antiaromatic nature of the imide ring. Although, NICS is a decent tool for interpretation of the aromaticity of a variety of cyclic compounds yet it has been criticized due to its unreliability to describe the aromaticity of the polycyclic structures.^28^ This is because NICS calculates the induced magnetic field at single point in space of a ring also affected by the σ-electrons while aromaticity is a property of global and local ring currents induced by a single ring and the rings associated with that ring.^29^

### AICD calculations

To obtain further insight into the ring current density as well as delocalization of the π-electron clouds, we performed AICD calculations.^30^ According to the AICD calculations, a high critical isosurface value (CIV) reflects strong delocalization of the π-electrons, and a low CIV represents weak conjugation at a critical point.^30b^ The CIVs have been assigned at the points of less conjugation for understanding the delocalization of electrons in both the dicationic and di-reduced systems. The AICD calculations of **1^2+^** and **2^2+^** show clockwise ring current on the naphthalene moiety indicating its aromatic nature, while in the imide region several of the current vectors were found to be disoriented which along with the anticlockwise current flow indicated antiaromatic nature of the imide rings (Fig. 6b and ESI Fig. S8b†).

The isosurface of the naphthalene annular bond of **1^2+^** gets broken at a CIV of 0.073 and for **2^2+^** it broke at 0.069 whereas, the side bonds rupture at 0.079–0.081 for **1^2+^** and 0.076–0.099 for **2^2+^**. These values confirm their diatropic nature. In contrast, in the imide part the C–N bond ruptures at 0.054 to 0.063 indicating less delocalization. The CIV value of the carbonyl group adjacent to the phosphonium group was found to be 0.092 for **1^2+^** and 0.089 for **2^2+^** which is significantly lower than the CIV (0.120 and 0.113) value of the carbonyl group remote to the phosphonium group, respectively. This clearly suggests the bond lengthening of carbonyl groups adjacent to the phosphonium group due to the strong P···O non-bonding interactions as validated from crystallographic studies (Fig. 6b).

On the other hand, in **1** and **2** the ring currents in the naphthalene unit are oriented in the anti-clockwise direction, which suggests antiaromaticity. In the imide rings, several of the current vectors were found to be disoriented along with current vectors oriented in a clockwise manner suggesting weakly aromatic nature (Fig. 6a and ESI Fig. S8a†). The isosurface of the naphthalene annular bond of **1** and **2** ruptures at a CIV value of 0.071 and 0.059 while the peripheral bonds were found to break at 0.062–0.072 and 0.059–0.072, respectively. These low CIV values of the naphthalene unit of di-reduced compounds showed slightly less electron delocalization compared to the dicationic molecules. There is a small increment in the CIV in the imide region in one of the C–N bonds and a small decrease in the CIV of other imide bonds in the di-reduced molecules. Furthermore, the CIV value of carbonyl group adjacent to phosphonium group was found to be 0.059 and 0.056 for **1** and **2**, respectively, while that of the carbonyl group remote to the phosphonium group was found to be 0.084 and 0.082. These CIV values are significantly lower than the CIV values found in the corresponding dicationic diphosphonium carbonyl groups. This corroborates lengthening in the bond lengths. Therefore, there is a swapping in the electron delocalization pattern between the naphthalene and the imide rings in both the dicationic and di-reduced systems.

### Current density maps and integral analysis

**Fig. 7:**
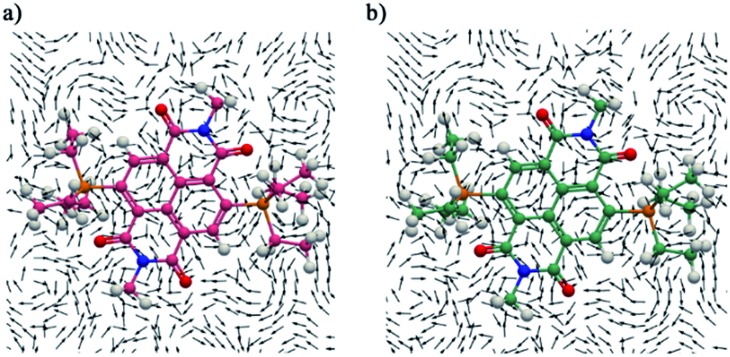
The magnetically induced current density of (a) **1** and (b) **1^2+^** calculated in a plane placed at 1.0 Bohr (1 Bohr = 0.529 Å) above the molecular plane. A magnet was placed along the *X* axis. Diatropic currents are assumed to circle clockwise and the paratropic ones circle anticlockwise.

The detailed information about the magnetically induced ring current densities,^31^ current strength and current pathways in both the direduced and dicationic compounds were investigated using the gauge-including magnetically induced current (GIMIC)^32^ method. The induced ring currents were visualized using ParaView 5.6.0.^33^ On careful examination of the ring currents it has been found that two weakly interacting ring currents; one corresponding to the diatropic ring current at the periphery of the molecule and one to the paratropic ring current in the center of the naphthalene ring of the NDI moiety circulate in both the direduced and dicationic compounds (Fig. 7 and ESI Fig. S9†).

To estimate the accurate ring current strength and current pathways we performed integration analysis of the key bonds by placing a plane at each of the bonds. The integration analysis of the current density passing through the plane of the C1–C2, C2–C3, C3–C4 and C4–C5 bonds in **1^2+^** shows a strong contribution of diatropic ring current of 16.37 to 17.97 nA T^–1^. It also shows a paratropic current contribution of –6.40 to –12.00 nA T^–1^ due to local current around carbon atoms and paratropic currents circling the naphthalene rings.

The integration of current strength passing a plane at the C5–C5′ bond yields a diatropic ring current of 13.04 nA T^–1^ and also a paratropic current contribution of 13.46 nA T^–1^. The net diatropic ring current of 4.74–11.6 nA T^–1^ in **1^2+^** validates the aromatic nature of the naphthalene ring (Table 3). The integration analysis of the C1–C6 bond corresponding to the imide region shows diatropic and paratrpoic ring currents of 6.76 nA T^–1^ and –19.57 nA T^–1^, respectively. A net current of –12.81 nA T^–1^ confirms antiaromatic nature, while the C6–N1, C7′–N1 and C4′–C7′ bonds associated with the imide ring show a diatropic current of 7.24–7.87 nA T^–1^ and paratropic contribution of –6.84 to –7.37 nA T^–1^. This corresponds to a net current of ∼0.5 nA T^–1^ and suggests non-aromatic nature. The net current of the C=O bonds of the dication also shows antiaromatic nature (Table 3).

**Table 3:** The diatropic, paratropic and net current strengths (current strength susceptibility in nA T^–1^) calculated at the B3LYP/6311++G(d,p) level. The numbering of the molecules is given in the figure

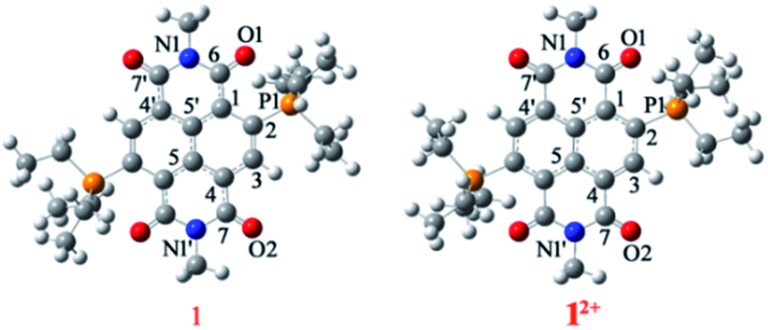						
Bond	Diatropic		Paratropic		Net current	
	**1**	**1^2+^**	**1**	**1^2+^**	**1**	**1^2+^**
C1–C2	8.76	17.97	–9.31	–6.40	–0.54	11.56
C2–C3	8.43	16.37	–17.57	–13.34	–9.13	3.03
C3–C4	8.17	17.13	–10.63	–7.71	–2.45	9.42
C4–C5	8.88	17.02	–16.70	–11.98	–7.81	5.03
C1–C5′	8.49	16.74	–16.44	–12.00	–7.94	4.74
C5–C5′	7.08	13.04	–16.03	–13.46	–8.95	–0.42
C1–C6	9.36	6.76	–17.92	–19.57	–8.56	–12.81
C4′–C7′	9.94	7.37	–4.45	–6.84	5.49	0.52
C6–N1	11.31	7.87	–5.73	–7.37	5.57	0.49
C7′–N1′	10.67	7.24	–5.24	–6.89	5.43	0.34
C6–O1	11.00	12.81	–18.36	–18.63	–7.35	–5.82
C7–O2	11.00	12.03	–16.05	–16.83	–5.04	–4.80
C2–P1	7.54	7.97	–6.51	–6.53	1.02	1.44

On the other hand, the diatropic ring current in C1–C2, C2–C3, C3–C4, C4–C5 and C5–C5′ bonds of **1** was found to be considerably moderated with values of 7.08–8.88 nA T^–1^, while the paratropic current contribution was found to be strong with a value of –9.31 to –17.57 nA T^–1^. The net current was found to be –0.54 to –8.95 nA T^–1^, which corroborates that this naphthalene ring possesses non-aromatic regions to antiaromatic regions (Table 3). The diatropic and paratropic ring contributions at the plane of the C1–C6 bond were found to be 9.36 nA T^–1^ and –17.92 nA T^–1^. This corresponds to a net current of –8.56 nA T^–1^, which suggests antiaromaticity at this position. However the C6–N1, C7′–N1 and C4′–C7′ bonds, which are also the part of the imide ring, yield diatropic 9.94–11.31 nA T^–1^ and paratropic –6.84 to –7.37 nA T^–1^ current contribution.

This leads to a net diatropic current with a value of 5.43 to 5.57 nA T^–1^ and suggests weak aromaticity in the imide ring in **1**. We also calculated the current contributions for **2** and **2^2+^** which are given in ESI Table S3.† The integration analysis also validated the switching of the aromatic and antiaromatic regions in the dicationic and the direduced compounds.

In essence, NICS, NICS-*XY* scan, AICD plots and GIMIC calculations suggest that there is a significant degree of switching in the aromaticity/antiaromaticity/nonaromaticity character in the naphthalene and the imide rings as a result of the two additional electrons in the di-reduced systems.

### FMO, ESP and NPA calculations

The ground state geometry optimization of **1** and **2** and **1^2+^** and **2^2+^** was performed by using the DFT/B3LYP method with the 6-311++G(d,p) basis set using the IEFPCM model in DCM. The axial groups in all the cases were replaced by the methyl group to save computational cost. The calculated HOMO and LUMO energies of **1** were found to be –4.145 and –1.436 eV, respectively, while for **2** they were –4.225 and –1.628 eV, respectively. For **1^2+^**, the HOMO and LUMO levels were –8.372 and –4.903 eV, respectively, and for **2^2+^** they were –8.063 and –4.894 eV, respectively. The calculated HOMO energy levels of di-reduced and LUMO energy levels of dications are in excellent correlation with electrochemically obtained HOMO–LUMO values (ESI Fig. S10†). The HOMO surface was found to be delocalized over the NDI moiety including the carbonyl oxygen in both **1** and **2**. The LUMO of **1** was predominantly delocalized over the NDI while in **2** it delocalizes over the two phosphonium groups with minor delocalization over the NDI unit. In the case of **1^2+^** and **2^2+^**, the LUMO was found to be delocalized over the NDI π-surface, while the HOMO was found to be delocalized over the phenyl phosphonium groups in **2^2+^** and over the NDI π-surface in **1^2+^**. The theoretically calculated HOMO and LUMO energy levels of **4**, **4^2+^**, **5** and **5^2+^** were also found to be in good agreement with the experimental data (ESI Fig. S8†).

**Fig. 8:**
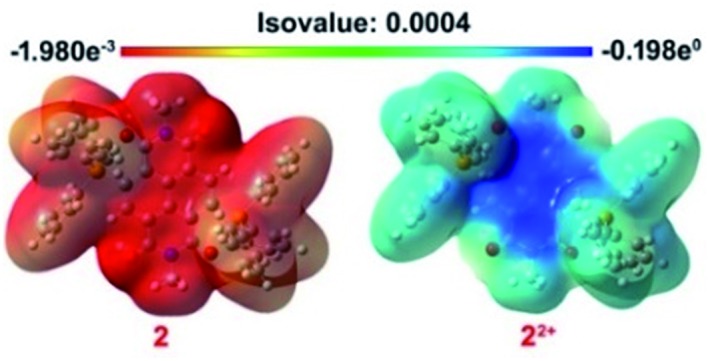
ESP maps of **2** and **2^2+^**. The contours are color-coded from red (electron-rich) to blue (electron-deficient).

The 3D electrostatic surface potential (ESP) distribution of **2^2+^** and **2** further supports the above findings. In **2^2+^**, a highly electron deficient region over the NDI surface is evident, while **2** shows a highly electron rich region delocalized over the NDI surface and also the triphenyl rings of the phosphonium groups (Fig. 8).

Natural population analysis (NPA)^34^ also revealed existence of higher negative charge on the oxygen atoms adjacent to phosphonium groups in the dicationic as well as in the di-reduced compounds compared to the oxygen atoms remote to phosphonium groups. Likewise, the phosphorus atoms retain most of the positive charge (ESI Table S4†).

### NBO analysis

Natural bond orbital (NBO) analysis^35^ shows the orbital interactions between the non-bonding orbitals of the imide oxygen atoms and the antibonding orbitals of the P–C bonds 
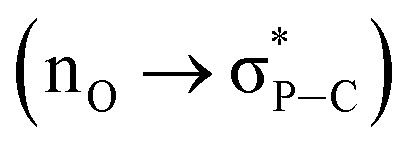
 (ESI Fig. S11†). A donor–acceptor type interaction with a stabilization energy of *E*_(2)_ = 2.24 kcal mol^–1^ for **2^2+^** and *E*_(2)_ = 4.44 kcal mol^–1^ for **2** per P···O interaction was estimated using second-order perturbation theory. This stronger P···O interaction in **2** further supports greater distribution of the negative charge through the imide carbonyl groups due to the additional two electrons in the di-reduced compounds.

### AIM analysis

Atoms in molecule (AIM) analysis^36^ also validate strong P···O interaction. An analysis of the topological properties of the electron density and determination of the bond critical point (BCP), which is accompanied by a bond path between the P atom of the phosphonium group and O atom of the imide carbonyl (P···O) was performed for **1**, **2** and its dicationic precursors.

**Fig. 9:**
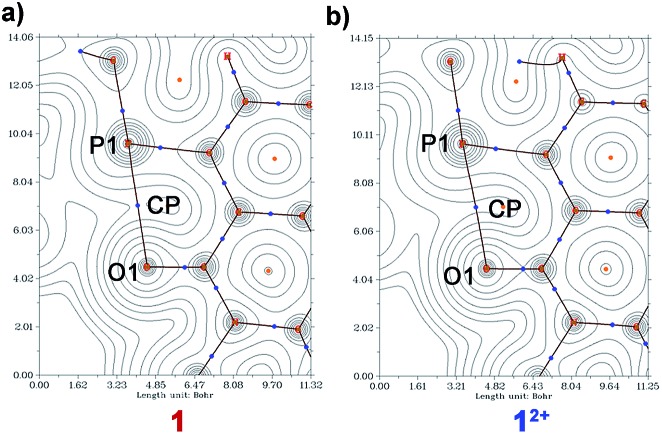
2D contour plots of (a) **1** and (b) **1^2+^** showing the bond path between P and O.

The 2D critical bond paths are shown in Fig. 9 and the corresponding 3D contour plots are shown in ESI Fig. S12†. The computed electron density parameters at the BCP, ∇^2^*ρ*(*r*_b_) P···O, *E*(*r*_b_)_P···O_, *G*(*r*_b_)_P···O_, and *V*(*r*_b_)_P···O_ for the P···O interactions are summarized in Table 3. The electron density, *ρ*(*r*_b_), values at the BCP of the P···O are in the range of H-bonding interactions (*ρ*_H_-bond ≈ 0.002–0.04 a.u.).^31^ The *ρ*(*r*_b_) for **1** (0.021 a.u.) and for **2** (0.025 a.u.) are higher than that for **1^2+^** (0.018 a.u.) and **2^2+^** (0.018 a.u.) validating a stronger P···O interaction in the di-reduced compounds. The Laplacian density, ∇^2^*ρ*(*r*_b_), is also in the range of strong H-bond interactions.

The positive 0.000 value of *E*(*r*_b_) for molecule **2^2+^** indicates its dominant electrostatic nature and the negative *E*(*r*_b_) values for **2** indicate its partial covalent nature [*E*(*r*_b_) value at a specific BCP indicates whether the interaction is electrostatic dominant *E*(*r*_b_) > 0 or covalent dominant *E*(*r*_b_) < 0]. The positive value of *G*(*r*_b_) and negative value of *V*(*r*_b_) support its stable, bound stationary states (Table 4). Inter-locked non-covalent interactions can thus play a significant role in electron delocalization.^37^

**Table 4:** AIM parameters for the dications and di-reduced compounds^*a*^

	Bond	*ρ*(*r*_b_)^*b*^	∇^2^*ρ*(*r*_b_)^*c*^	*E*(*r*_b_)^*d*^	*G*(*r*_b_)^*e*^	*V*(*r*_b_)^*f*^
**1**	P···O	0.021	0.051	–0.001	0.013	–0.014
**1^2+^**	P···O	0.018	0.050	–0.000	0.012	–0.013
**2**	P···O	0.025	0.055	–0.001	0.015	–0.016
**2^2+^**	P···O	0.018	0.051	0.000	0.012	–0.012

^*a*^Theoretical topological properties at the BCP; values from the 6-311++G(d,p) basis set.

^*b*^The electron density at the BCP.

^*c*^Laplacian of electron density.

^*d*^The electron energy density.

^*e*^Kinetic energy electron density.

^*f*^Potential energy electron density. All are in a.u.

## Conclusions

We demonstrated isolation of two-electron (2e^–^) reduced, highly electron-rich, bench-stable NDIs. A doubly zwitterionic structure is observed in a naphthalene moiety and validated by single crystal X-ray crystallography. This new genre of compounds was achieved in high-yields *via* a solvent-free synthetic protocol. The di-reduced systems endow exceptional inherent stability with a half-life time of more than six months in toluene under ambient conditions. Notably, high negative first oxidation potentials up to –0.730 V *vs.* Fc/Fc^+^ and HOMO levels extending to –4.360 eV were realized.

The study offers the first insights into the NMR spectra of the di-reduced systems revealing large upfield chemical shifts for the NDI-core atoms and the adjoining groups compared to their 2e^–^ oxidized form, suggesting a large decrease in the diatropicity of the naphthalene ring. The NICS, NICS-*XY*, GIMIC and AICD calculations revealed redox switching of the antiaromatic and aromatic states at the naphthalene and the imide rings, respectively, in the di-reduced system compared to the corresponding rings of the 2e^–^ oxidized form. The substituents at the axial- and core-positions tune the antiaromatic–aromatic states and the electron donor ability. Further an array of diverse colors was achieved for the di-reduced systems. The through-space non-covalent interactions are key elements assisting this tunability. Isolation of this new class of ambient stable systems should have fascinating implications in controlling electron transfer reactions and development of new switchable materials.

## Conflicts of interest

There are no conflicts to declare.

## Supplementary Material

Supplementary information

Crystal structure data
