# Supplementary material for: Doubly zwitterionic, di-reduced, highly electron-rich, air-stable naphthalenediimides: redox-switchable islands of aromatic–antiaromatic states
Source: Chem Sci. 2019 May 21;10(26):6482–93. doi: 10.1039/c9sc00962k (PMC6611073; doi:10.1039/c9sc00962k)
Supplement: Supplementary file 1 [file SC-010-C9SC00962K-s001.pdf]

Electronic Supplementary Material (ESI) for Chemical Science.
This journal is © The Royal Society of Chemistry 2019

Doubly-Zwitterionic, Di-reduced, Highly Electron-Rich, Air-Stable Naphthalenediimides: Redox-Switchable Islands of Aromatic-Antiaromatic States

Sharvan Kumar,[a] Jyoti Shukla,[a] Kalyanashis Mandal,[a] Yogendra Kumar,[a] Ravi Prakash,[b] Panch Ram,[b] and Pritam Mukhopadhyay[a]*.

[a]*Supramolecular and Material Chemistry Lab, School of Physical Sciences, Jawaharlal Nehru University, New Delhi–110067, India; E-mail: m_pritam@mail.jnu.ac.in.*

[b]*School of Physical Sciences, Jawaharlal Nehru University, New Delhi–110067, India.*

Table of Contents

1. Theoretical and Experimental Details……..…………………………………. S2
2. Synthesis……………………………………………………………….... S4
3. UV-Vis Spectra of Reaction Crude Product ………………………………. S8
4. UV-Vis Spectra of Dication Reaction with Et_3N……………………………… S9
5. Plausible Reaction Mechanism……………………………………………… S9
6. Comparison of the Selected Bond lengths and Torsion Angles of **1** and **1^{2+}. 2BF$_4^-$** (Crystal Structures with Optimized Structures) ………………………………… S10
7. Comparison of the Selected Bond lengths and Torsion Angles of **2** and **2^{2+}. 2BF$_4^-$** (Crystal Structures with Optimized Structures) ………………………………… S11
8. CV and DPV of Compound **1^{2+}-5^{2+}** ……………………………………… … S12
9. DPV of Direduced **1-5** ……………………………………………………… S13
10. Square root of Scan Rate vs Current Peak………………………………. S14
11. UV-Vis Spectra Showing the Direduced formation by Reduction of Radical Ion.. S15
12. UV-Vis Spectra Showing the Stability of **4** in Different Solvents …………. ….. S15
13. AICD Isosurface Plots of a) **2**, and b) **2^{2+}** ……………………………......... S16
14. The Magnetically Induced Current Density of a) **2**, and b) **2^{2+}**…………………… S16
15. Table Integration Analysis of molecule **2** and **2^{2+}**……………………………….. S17
16. HOMO-LUMO Diagram ……………………….……………………….. S18
17. Table of Natural Population Analysis……………………………………. S19
18. NBO plots of **2** and **2^{2+}**………………………………………………….... S19
19. 3D-Critical Path (AIM) Analysis……………………………………….. S20
20. Table of crystal Data **1**, **1^{2+}**, **2** and **2^{2+}** ………………..………………….….... S21
21. FT-IR-Spectra……………………………………………………………... S22
22. Mass Spectrometry………………………………………………………... S23
23. NMR………………………………………………………………………. S25
24. References………………………………………………………………… S39

Theoretical Details: The ground-state geometry optimization of the investigated structures was carried out in gas phase at the Becke three-parameter[1] hybrid exchange functional in concurrence with the Lee-Yang-Parr gradient-corrected correlation function (B3LYP functional)[2] level of the density functional theory (DFT), using the 6-311++G(d,p) basis set for **1** and **2** molecules. DFT calculations have been performed on all stationary points of the potential energy surface (PES) and studied using the Gaussian 09W.[3] The geometries were optimized without any constrain. To reduce the calculation time molecule's axial group (R′) have been replaced by methyl group and the dications were taken without counter anion. The electrostatic potentials (ESP) mapped on the electronic density surfaces and HOMO-LUMO analysis were based on the DFT calculations and plotted with the Gauss View 5.0.9 program.

NICS and NICS-XY calculations were carried out using the above-mentioned optimized structure as input file and performed in same Gaussian 09W. The NICS-XY scans were carried out using the Aroma package described by Stanger and co-workers[4] at the level of theory mentioned above. ^{1}H NMR were calculated using optimized structure in Gaussian 09W using NMR=GIAO key word. The same optimized structure were used as input file for AICD calculations and to obtain the AICD input file for calculation of the anisotropy of the current density; calculations were performed in Gaussian 09W program using NMR=CSGT with a special key word (IOp(10/93=1). The anisotropy of current density was plotted using AICD 2.0.0 package described and provided by Dr. R. Herges.[5] Magnetically induced current densities were calculated at the B3LYP/def2-TZVP level using the GIMIC program,[6] which is a free-standing program employed to calculate current densities. GIMIC uses the atomic orbital density matrix and the first-order magnetically perturbed density matrices as well as basis-set information as input data.[6] The density matrices are obtained in the electronic structure calculation and in the calculation of nuclear magnetic shielding constants, respectively. Gauge-origin independence and a fast basis-set convergence of the current densities are ensured by using GIAOs.[7] Graphical visualization was done using Paraview.[8]

The natural bond orbital (NBO) population analysis was performed with Weinhold's methodology[9] using the above mentioned optimized structures as input. Further the atoms in molecule (AIM)[10] and electron localization function (ELF)[11] calculations were performed with the Multiwfn 3.3.7 (dev) package[12] using the DFT/RB3LYP/6311+G(d,P) or 631+G(d,p) optimized structures as input.

Experimental Details

General: All the starting materials were sourced either from Sigma-Aldrich, TCI, Spectrochem (India), Loba Chemie (India) or Thomas Baker (India). Triethylamine (spectrochem) was dried and triphenylphosphine (sepctrochem) was recrystallized prior to use; rest chemicals were used as received. The reactions for the synthesis of *in situ* direduced and their parent compounds viz. radical ions were carried out in heavy-wall borosilicate glass tubes (L x OD: 17.8 cm x 25.4 mm) sourced from Sigma Aldrich (Cat. No. Z181072). Thin layer chromatography (TLC) was carried out on aluminium plates coated with silica gel mixed with fluorescent indicator and was sourced from Merck, Germany. NMR (^{1}H, ^{13}C, DEPT-135 and APT) spectra were recorded on a Bruker 500 MHz spectrometer in $CD_2Cl_2/CDCl_3$ with TMS as a standard. ^{31}P NMR was performed in Bruker 500 MHz spectrometer in CD_2Cl_2/ $CDCl_3$ with H_3PO_4 (70% aqueous solution) as an external standard. Spin multiplicities are reported as a singlet (s), doublet (d), and triplet (t) with coupling constants (*J*) given in Hz, or multiplet (m). MALDI-TOF mass spectral data were obtained using a Bruker made Autoflex TOF/TOF instrument with laser repetition rate of 50 psec. 1,8,9-Anthracenetriol was used as the matrix for MALDI-TOF mass spectrometry.

UV-Vis and FT-IR Spectroscopy: UV-Vis-NIR spectra were recorded on a JASCO V-670 Spectrophotometer. All UV-Vis-NIR spectroscopic experiments were performed in a quartz cuvette with 10.0 mm optical pathlength. UV-Grade solvents were used for the spectroscopic experiments. Wavelength reported in nanometres (nm). Fourier transform infrared (FTIR) spectra were recorded on Varian 7000 FT-IR spectrometer. A pellet of samples were prepared in a dry, finally ground KBr matrix for data collection. A blank scan was run to cut out the air effects before analysis.

Cyclic and Differential Pulse Voltammetry (CV/DPV): CV and DPV were carried out using a computer controlled potentiostat (CHI 650C) and a standard three electrode arrangement that consisted of both platinum working and auxiliary electrodes and Ag/AgCl as reference electrode. All the electrochemical measurements were carried out in Ar-purged solvents with n-Bu_4NPF_6 as the supporting electrolyte. The scan rate for the measurements were typically 200-300 mV/s. DPV was carried out keeping peak amplitude 50 mV, peak width 0.01 sec, pulse period 0.05 sec and increment E at 20 mV.

X-Ray Crystallography: Crystals of $\mathbf{1}^{2+}$ and $\mathbf{2}^{2+}$ were grown in Toluene/CH_2Cl_2 solution by slow evaporation method at room temperature and **1** and **2** crystals were grown in Toluene/CH_2Cl_2 solution by slow evaporation method in refrigerator. The $\mathbf{1}^{2+}$ and $\mathbf{2}^{2+}$ crystals

were highly stable under ambient conditions while **1** and **2** crystals were kept in paratone oil just after taking from mother liquid. The reported data set was collected by mounting the crystal with paratone oil on the loop at 100 K. The X-ray data were collected on the Bruker APEX-II CMOS diffractometer using Mo-Kα radiation (λ = 0.71073 Å), generated from the micro-focus sealed tube using φ and ω-scans of 0.5° steps at 100 K. Cell determination, data collection and data reduction were performed with the help of Bruker APEX2 (version: 2014.3-0) software. Structure solution and refinement were performed using SHELXS-97 incorporated in the WinGX software interface. Refinement of coordinates and anisotropic thermal parameters of non-hydrogen atoms were carried out by the full-matrix least-squares method. The hydrogen atoms were generated with idealized geometries and refined isotropically using a riding model.

General Procedure for Realization of *in situ* Synthesis of 3-5: In a pressure tube (for details, see General Experimental Section) corresponding axially substituted NDI-$(Br)_2$, 4.0 equivalent phosphine and 1.5 equivalent $(C_2H_5)_3N$ was added. The reaction mixture was allowed to heat at 100 °C on a magnetic stirrer. The colour of the reaction mixture became brown (in case of **5** blue) with the progress of the reaction. After 30 min, the reaction mixture was gradually brought to room temperature. However their purification in direduced form was not realized due to instability of direduced molecules on silica-gel. They have been purified in corresponding radical ion viz. **$2^{\cdot+}$-$5^{\cdot+}$** form as a dark green colour compound by silica-gel column chromatography (100-200 mesh) with $CHCl_3$/MeOH (100:1) as the eluent.

Synthesis of 1-5 Using $1^{\cdot+}$-$5^{\cdot+}$ Radical Ions

Typical Procedure: In a stirring solution of radical ions in DCM, a solution of Na_2S in MeOH was added drop wise until the colour of the solution gets converted from green to brown. The reaction mixture was stirred at room temperature for 15 minutes. The reaction progress was monitored by UV-Vis spectroscopy. The complete disappearance of radical ion characteristic peaks and appearance of direduced characteristic peaks denote the completion of reaction. If not, further Na_2S was added. Reaction mixture was dried washed with hexane dissolved again in DCM and filtered. The filtrate was vacuum dried to get a pure dark brown solid of **1-5**. Isolated yield ~70-80 %.

Direduced 1: $1^{\bullet+}$ (200 mg, 0.29 mmol), yield ~70 %. M. P.: 230 °C. ^{1}H NMR (500 MHz, CD_2Cl_2, 298 K, TMS): δ (ppm) = 7.33 (d, *J* = 17 Hz, 2H, *Nap*), 7.28 (d, *J* = 7.0 Hz, 4H), 7.25 (t, *J* = 7.5 Hz, 4H), 7.16 (t, *J* = 7.0 Hz, 2H), 5.32 (s, 4H), 2.34 (b, 12H), 1.18-1.08 (m, 18H). ^{13}C NMR (125 MHz, CD_2Cl_2, 298 K, TMS): δ (ppm) =159.73, 139.44, 137.96, 134.56, 128.96,

128.15, 127.95, 127.58, 126.35, 125.23, 77.58, 58.08, 43.18, 21.15, 16.78, 6.65, 6.62. APT (125 MHz, CD_2Cl_2, 298 K, TMS): δ (ppm) = 159.73, 139.44, 137.96, 128.96, 128.15, 127.95, 127.58, 126.35, 125.23, 58.08, 43.18, 21.15, 6.65, 6.62. DEPT-135 (125 MHz, CD_2Cl_2, 298 K, TMS): δ (ppm) = 128.96, 128.15, 127.95, 127.58, 126.35, 125.23, 58.08, 43.18, 21.15, 6.65, 6.62.^{31}P (202 MHz, CD_2Cl_2, 298 K, H_3PO_4): δ (ppm) = 32.90. (MALDI-TOF matrix: 1,8,9-Anthracenetriol): calculated for $C_{40}H_{46}N_2O_4P_2$ (m/z) 680.29, found 680.16. FT-IR (KBr pellet, cm^{-1}): 3055, 2973, 2938, 2883, 1648, 1617, 1519, 1458, 1307, 1213, 1133, 1044.

Direduced 2: 2$^{\bullet+}$ (200 mg, 0.18 mmol), yield ~75 %. M. P.: 220 °C. ^{1}H NMR (500 MHz, CD_2Cl_2, 298 K, TMS): δ (ppm) = 7.79-7.56 (m, 34H), 6.97 (b, 4H), 2.21 (b, 4H) 0.94 (d, *J* = 6 Hz, 12H) 0.63 (d, *J* = 6 Hz, 12H).^{13}C NMR (125 MHz, CD_2Cl_2, 298 K, TMS): δ (ppm) =134.94, 132.54, 131.88, 128.97, 128.86, 128.67, 127.68, 123.11, 29.04, 28.38, 23.63, 23.56, 11.19. APT (125 MHz, CD_2Cl_2, 298 K, TMS): δ (ppm) = 132.52, 131.87, 128.96, 128.59, 127.67, 123.10, 28.38, 23.63, 23.56. DEPT-135 (125 MHz, CD_2Cl_2, 298 K, TMS): δ (ppm) = 132.52, 131.87, 128.96, 128.59, 127.67, 123.10, 28.38, 23.63, 23.56. ^{31}P (202 MHz, CD_2Cl_2, 298 K, H_3PO_4): δ (ppm) = 25.45. (MALDI-TOF matrix: 1,8,9-Anthracenetriol): calculated for $C_{74}H_{66}N_2O_4P_2$ (m/z) 1108.45, found 1108.39. FT-IR (KBr pellet, cm^{-1}): 3058, 2960, 2928, 2930, 2867, 1630, 1560, 1457, 1435, 1213, 1105, 1023.

Direduced 3: 3$^{\bullet+}$ (100 mg, 0.12 mmol), yield ~80 %. M. P.: 248 °C. ^{1}H NMR (500 MHz, $CDCl_3$, 298 K, TMS): δ (ppm) = 7.50-7.44 (m, 8H), 7.40 (d, *J* = 17.5 Hz, 2H) 5.43 (s, 4H), 2.35 (b, 12H) 1.15 (m, 18H).^{13}C NMR (125 MHz, $CDCl_3$, 298 K, TMS): δ (ppm) =143.10, 129.08, 128.83, 128.57, 128.32, 128.04, 127.60, 125.43, 125.05, 125.02, 124.99, 123.27, 121.11, 43.28, 22.67, 17.12, 6.85. APT (125 MHz, $CDCl_3$, 298 K, TMS): δ (ppm) = 129.08, 128.83, 128.57, 128.32, 128.04, 127.60, 125.43, 125.05, 125.02, 124.99, 123.27, 121.11, 43.28, 22.67, 17.12, 6.85. DEPT-135 (125 MHz, $CDCl_3$, 298 K, TMS): δ (ppm) = 128.04, 125.05, 125.02, 124.99, 43.28, 6.85. ^{31}P (202 MHz, $CDCl_3$, 298 K, H_3PO_4): δ (ppm) = 33.25. (MALDI-TOF matrix: 1,8,9-Anthracenetriol): calculated for $C_{42}H_{44}F_6N_2O_4P_2$ (m/z) 816.27, found 816.33. FT-IR (KBr pellet, cm^{-1}): 3053, 2976, 2941, 2883, 1619, 1556, 1454, 1418, 1325, 1205, 1163, 1113, 1068, 1043.

Direduced 4: 4$^{\bullet+}$ (150 mg, 0.13 mmol), yield ~70 %. M. P.: 290 °C. ^{1}H NMR (500 MHz, $CDCl_3$, 298 K, TMS): δ (ppm) = 7.78-6.86 (m, 40H), 4.94 (s, 4H).^{13}C NMR (125 MHz, $CDCl_3$, 298 K, TMS): δ (ppm) =159.82, 159.18, 142.66, 140.43, 140.32, 131.97, 128.88, 128.30, 126.72, 124.60, 104.10, 104.04, 97.28, 96.93, 42.65, 30.97. APT (125 MHz, $CDCl_3$, 298 K,

TMS): δ (ppm) = 159.82, 159.18, 142.66, 141.70, 140.43, 140.32, 131.97, 128.88, 128.30, 126.72, 124.60, 104.10, 104.04, 97.28, 96.93, 42.65. DEPT-135 (125 MHz, $CDCl_3$, 298 K, TMS): δ (ppm) = 140.43, 140.32, 131.97, 128.88, 128.30, 126.72, 124.60, 124.57, 42.65, 30.97. ^{31}P (202 MHz, $CDCl_3$, 298 K, H_3PO_4): δ (ppm) = 25.87. (MALDI-TOF matrix: 1,8,9-Anthracenetriol): calculated for $C_{66}H_{44}F_6N_2O_4P_2$ (m/z) 1104.27, found 1104.25. FT-IR (KBr pellet, cm^{-1}): 3061, 2963, 2933, 2858, 1622, 1559, 1520, 1439, 1418, 1325, 1163, 1211, 1112.

Direduced 5: $\mathbf{5^{\cdot+}}$ (50 mg, 0.03 mmol), yield ~30 %. M. P.: >300 °C. ^1H NMR (500 MHz, CD_2Cl_2, 298 K, TMS): δ (ppm) = 7.91-7.71 and 7.34 (m, 26H), 6.70 (s, 4H), 2.16(s, 6H), 1.50(s, 12H). ^{13}C NMR could not observed due to low solubility. ^{31}P (202 MHz, CD_2Cl_2, 298 K, H_3PO_4): δ (ppm) = 24.89. (MALDI-TOF matrix: 1,8,9-Anthracenetriol): calculated for $C_{74}H_{48}F_{18}N_2O_4P_2$ (m/z) 1432.28, found 1432.24. FT-IR (KBr pellet, cm^{-1}): 3099, 3039, 2963, 2924, 2858, 1635, 1553, 1424, 1323, 1219, 1174, 1063.

Synthesis of $\mathbf{1^{2+}}$-$\mathbf{5^{2+}}$: To compare the direduced compounds data with dicationic one, corresponding dications were synthesized. The dicationic compounds $\mathbf{1^{2+}}$-$\mathbf{5^{2+}}$ were synthesized by oxidizing $\mathbf{1^{\cdot+}}$-$\mathbf{5^{\cdot+}}$ or direduced **1-5** with $NOBF_4$ in $CHCl_3$ at room temperature as $\mathbf{1^{2+}.\ 2BF_4^-}$-$\mathbf{5^{2+}.2BF_4^-}$.

Typical Procedure: In a stirring solution of radical ions/direduced in $CHCl_3$, 1.5/3.0 equivalent of $NOBF_4$ was added and the reaction mixture was stirred at room temperature for 15 minutes. The colour of the solution gets converted from green or brown to yellow. After that, the reaction mixture was dried and purified by silica gel (100-200 mesh) column chromatography using $CHCl_3$: MeOH (99:1) as eluent or by recrystallization method.

$\mathbf{1^{2+}.\ 2BF_4^-}$**:** $\mathbf{1^{\cdot+}}$ (200 mg, 0.26 mmol) and $NOBF_4$ (46.0 mg, 0.39 mmol), and $CHCl_3$ (7.0 mL), Yield = 80%. R_f = 0.50 (9:1 $CHCl_3$/MeOH). M. P.: 275 °C. ^1H NMR (500 MHz, CD_2Cl_2, 298 K, TMS): δ (ppm) = 8.94 (d, J = 11.0 Hz, 2H), 7.48 (d, J = 7.5 Hz, 4H), 7.34 (t, J = 7.5 Hz, 4H), 7.29 (t, J = 7.0 Hz, 2H) 5.41 (s, 4H, NCH_2), 2.75 (m, 12H), 1.30 (m, 18H).^{13}C NMR (125 MHz, CD_2Cl_2, 298 K, TMS): δ (ppm) =163.63, 161.01, 135.46, 134.53, 134.46, 131.76, 128.94, 128.65, 128.39, 128.13, 127.96, 125.83, 125.23, 44.99, 15.14, 14.73. APT (125 MHz, CD_2Cl_2, 298 K, TMS): δ (ppm) = 163.63, 161.01, 135.46, 134.53, 134.46, 131.76, 128.94, 128.65, 128.39, 128.13, 127.96, 125.83, 125.23, 44.99, 15.14, 14.73. DEPT-135 (125 MHz, CD_2Cl_2, 298 K, TMS): δ (ppm) = 134.53, 134.46, 131.76, 128.94, 128.65, 128.39, 128.13, 127.96, 125.83, 125.23, 44.99, 15.14, 14.73. ^{31}P (202 MHz, CD_2Cl_2, 298 K, H_3PO_4): δ (ppm) = 44.07. MS (MALDI-TOF matrix: 1,8,9-Anthracenetriol): Calculated for $C_{40}H_{46}N_2O_4P_2$

$[M{-}2BF_4^-]^{2+}$ 680.29, found 680.16. FT-IR (KBr pellet): $\bar{\nu}$ (cm^{-1}) = 2980, 2935, 2904, 1715, 1667, 1561, 1542, 1443, 1375, 1325, 1221, 1048.

$\mathbf{2^{2+}.\ 2BF_4^-}$**:** $\mathbf{2^{\bullet+}}$ (300 mg, 0.25 mmol) and $NOBF_4$ (44.0 mg, 0.38 mmol), and $CHCl_3$ (10.0 mL), Yield = 80%. R_f = 0.51 (8.5:1.5 $CHCl_3$/MeOH). M. P.: >300 ˚C. ^{1}H NMR (500 MHz, CD_2Cl_2, 298 K, TMS): δ (ppm) = 8.42 (d, *J* = 13.0 Hz, 2H), 7.89-7.64 (m, 30H, PPh_3), 7.33 (d, *J* = 7.5 Hz, 2H), 7.13 (d, *J* = 7.5 Hz, 4H) 2.53 (b, 4H), 0.95 (b, 12H), 0.64 (b, 12H). ^{13}C NMR (125 MHz, CD_2Cl_2, 298 K, TMS): δ (ppm) =162.61, 161.20, 146.22, 137.58, 137.48, 134.52, 132.67, 130.41, 130.30, 129.93, 129.09, 125.47, 124.01, 28.51, 23.83, 23.79. APT (125 MHz, CD_2Cl_2, 298 K, TMS): δ (ppm) = 162.61, 161.20, 146.22, 137.58, 137.48, 134.52, 132.67, 130.41, 130.30, 129.93, 129.09, 125.47, 124.01, 28.51, 23.83, 23.79. DEPT-135 (125 MHz, CD_2Cl_2, 298 K, TMS): δ (ppm) = 137.58, 137.48, 134.52, 132.67, 130.41, 130.30, 129.93, 129.09, 124.01, 28.51, 23.83, 23.79. ^{31}P (202 MHz, CD_2Cl_2, 298 K, H_3PO_4): δ (ppm) = 31.09. MS (MALDI-TOF matrix: 1,8,9-Anthracenetriol): Calculated for $C_{74}H_{66}N_2O_4P_2$ $[M{-}2BF_4^-]^{2+}$ 1108.45, found 1108.44. FTIR (KBr pellet): $\bar{\nu}$ (cm^{-1}) = 3057, 2961, 2929, 2866, 1701, 1660, 1613, 1560, 1515, 1461, 1314, 1211, 1106.

$\mathbf{3^{2+}.\ 2BF_4^-}$**:** (100 mg, 0.12 mmol), yield ~80 %. R_f = 0.50 (9:1 $CHCl_3$/MeOH). M. P.: 248 °C. ^{1}H NMR (500 MHz, $CDCl_3$, 298 K, TMS): δ (ppm) = 8.89 (d, *J* = 11.0 Hz, 2H), 7.60 (b, 8H), 5.44 (s, 4H, NCH_2), 2.75 (b, 12H) 1.31 (m, 18H).^{13}C NMR could not be performed due to low solubility. ^{31}P (202 MHz, $CDCl_3$, 298 K, H_3PO_4): δ (ppm) = 43.53. (MALDI-TOF matrix: 1,8,9-Anthracenetriol): calculated for $C_{42}H_{44}F_6N_2O_4P_2$ (m/z) 816.27, found 816.33. FT-IR (KBr pellet, cm^{-1}): 3053, 2976, 2941, 2883, 1619, 1556, 1454, 1418, 1325, 1205, 1163, 1113, 1068, 1043.

$\mathbf{4^{2+}.\ 2BF_4^-}$**:** $\mathbf{4^{\bullet+}}$ (200 mg, 0.17 mmol) and $NOBF_4$ (29.0 mg, 0.25 mmol), and $CHCl_3$ (7.0 mL), Yield = 80%. R_f = 0.60 (8.5:1.5 $CHCl_3$/MeOH). M. P.: >300 ˚C. ^{1}H NMR (500 MHz, $CDCl_3$, 298 K, TMS): δ (ppm) = 8.35 (d, *J* = 14.0 Hz, 2H), 7.70-7.58 (m, 30H, PPh_3), 7.33 (d, *J* = 8.5 Hz, 4H), 7.00 (d, *J* = 8.0 Hz, 4H) 3.77 (s, 4H, NCH_2).^{13}C NMR (125 MHz, $CDCl_3$, 298 K, TMS): δ (ppm) =161.95, 160.85, 139.10, 136.94, 136.85, 134.53, 133.63, 130.31, 130.20, 127.71, 125.30, 125.28, 65.89, 43.56. APT (125 MHz, $CDCl_3$, 298 K, TMS): δ (ppm) = 161.95, 160.85, 139.10, 136.94, 136.85, 134.53, 133.63, 130.31, 130.20, 127.71, 125.30, 125.28, 65.89, 43.56. DEPT-135 (125 MHz, $CDCl_3$, 298 K, TMS): δ (ppm) = 133.63, 130.31, 130.20, 127.71, 125.30, 125.28, 65.89, 43.56. ^{31}P (202 MHz, $CDCl_3$, 298 K, H_3PO_4): δ (ppm) = 31.12. MS (MALDI-TOF matrix: 1,8,9-Anthracenetriol): Calculated for $C_{66}H_{44}F_6N_2O_4P_2$ $[M{-}2BF_4^-]^{2+}$

1104.27, found 1104.26. FTIR (KBr pellet): $\bar{\nu}$ (cm^{-1}) = 3068, 2970, 2859, 1719, 1699, 1658, 1541, 1442, 1326, 1112, 1066.

5^{2+}. $2BF_4^-$: (50 mg, 0.03 mmol), yield ~50 %. R_f = 0.54 (8.5:1.5 $CHCl_3$/MeOH). M. P.: >300 °C. ^{1}H NMR (500 MHz, CD_2Cl_2, 298 K, TMS): δ (ppm) = 8.45 (d, *J* = 14.5 Hz, 2H), 8.07-7.96 (m, 24H, *p*-CF_3PPh_3), 6.84 (s, 4H), 2.22 (s, 6H) 1.58 (s, 12H).^{13}C NMR could not be performed due to low solubility. ^{31}P (202 MHz, CD_2Cl_2, 298 K, H_3PO_4): δ (ppm) = 30.45. (MALDI-TOF matrix: 1,8,9-Anthracenetriol): calculated for $C_{74}H_{48}F_{18}N_2O_4P_2$ (m/z) 1432.28, found 1432.24. FT-IR (KBr pellet, cm^{-1}): 3099, 3039, 2963, 2924, 2858, 1635, 1553, 1424, 1323, 1219, 1174, 1063.

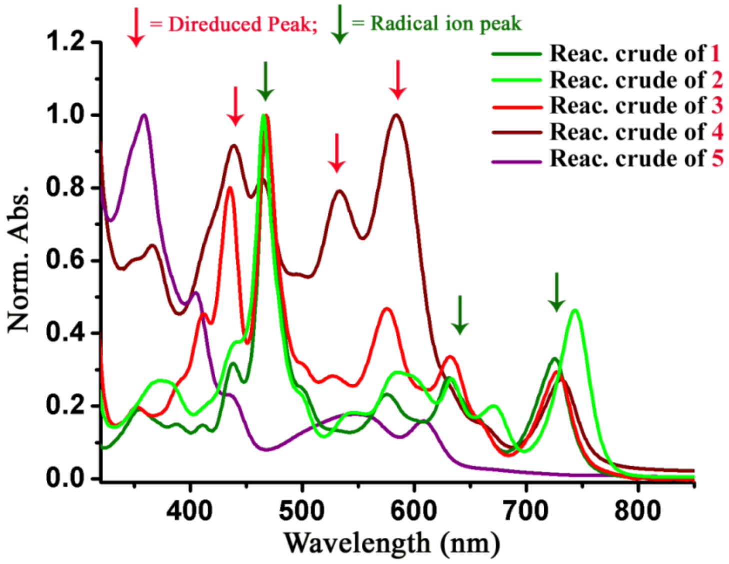

Figure S1: UV-Vis-NIR spectra of the crude reaction products of **1-5** in DCM. This shows the formation of only radical ion for compound **1**, formation of direduced as well as radical ions for **2-4** and formation of direduced compound with some other product in case of **5**.

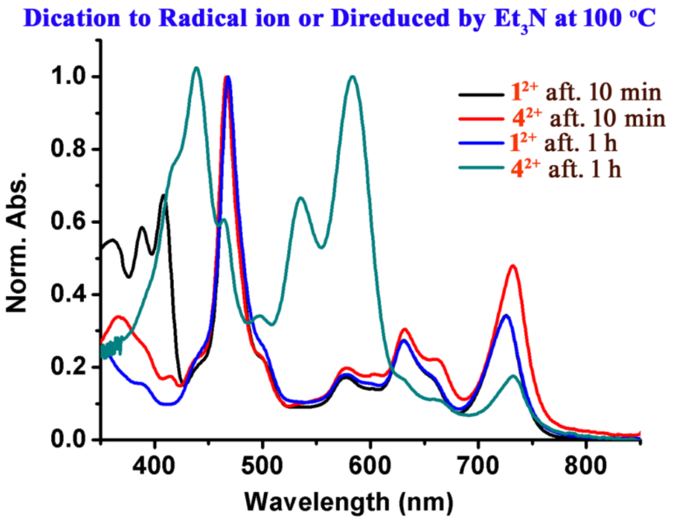

Figure S2: Normalized UV-Vis-NIR spectra showing the response of dication $\mathbf{1^{2+}. 2BF_4}$ and $\mathbf{4^{2+}. 2BF_4}$ reaction with triethylamine at 100 °C in DCM. This shows the formation of only radical ion for compound $\mathbf{1^{2+}. 2BF_4}$, and formation of direduced for $\mathbf{4^{2+}. 2BF_4}$.

Scheme 1: Plausible reaction Mechanism of formation of in situ radical ions and direduced compounds.

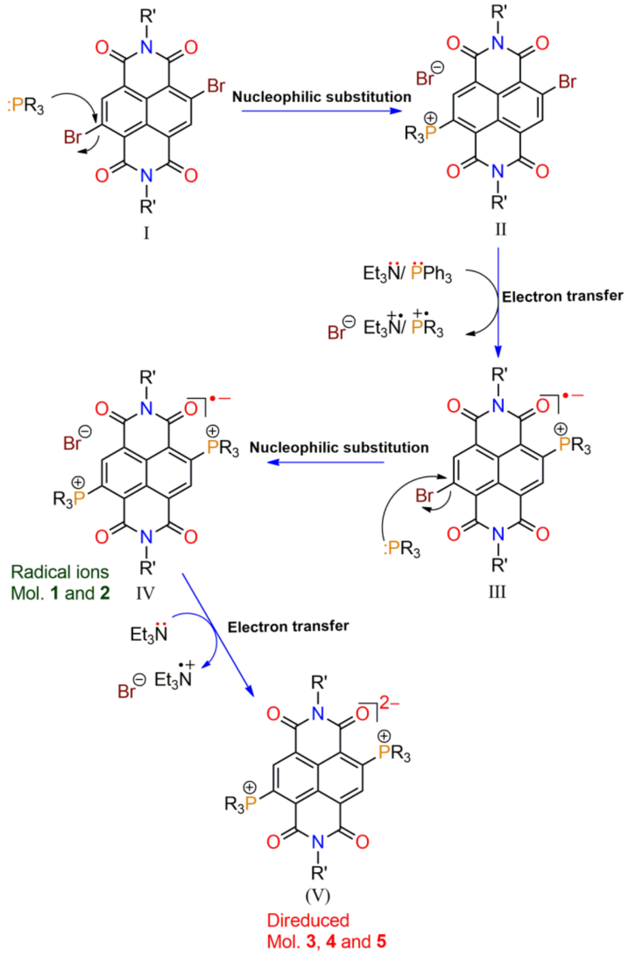

Table S1: A comparison of the selected bond lengths (Å) and torsional angles (°) of X-ray crystal structures of **1** and $\mathbf{1}^{2+}$ and the geometry optimized structures of **1** and $\mathbf{1}^{2+}$.

Bond	Crystal data		Theor. Data	
	**1**	$\mathbf{1}^{2+}$	**1**	$\mathbf{1}^{2+}$
C1-C2, C1’-C2’	1.456 (4), 1.453	1.396 (2)	1.456, 1.454	1.399
C2-C3, C2’-C3’	1.371 (4), 1.374	1.413 (2)	1.378	1.414
C3-C4, C3’-C4’	1.423 (4), 1.417	1.373 (2)	1.411, 1.422	1.379
C4-C5, C4’-C5’	1.409 (4), 1.413	1.410 (2)	1.417, 1.418	1.409
C1-C5’, C1’-C5	1.405 (4), 1.414	1.407 (2)	1.408, 1.409	1.418
C5-C5’	1.437 (4)	1.410 (2)	1.438	1.418
C1-C6, C1’-C6’	1.431 (4), 1.430	1.489 (2)	1.431, 1.429	1.491
C4-C7, C4’-C7’	1.434 (4), 1.431	1.483 (2)	1.445, 1.446	1.491
C6-N1, C6’-N1’	1.394 (4), 1.403	1.392(2)	1.392	1.382
C7-N1’, C7’-N1	1.406 (4), 1.402	1.386(2)	1.413, 1.414	1.404
C6-O1, C6’-O1’	1.252 (4), 1.244	1.212(2)	1.255	1.221
C7-O2, C7’-O2’	1.244 (4), 1.236	1.219(1)	1.235	1.208
N1-C8, N1’-C8’’	1.465 (3), 1.466	1.478 (2)	1.464	1.479
C2-P1, C2’-P2’	1.795 (3), 1.791	1.827(1)	1.793, 1.800	1.862, 1.869
P1-C17, P1’-C17’	1.809 (3), 1.811	1.802 (2)	1.845	1.841
P1-C15, P1’-C15’	1.813 (3), 1.820	1.815 (1)	1.851	1.842
P1-C19, P1’-C19’	1.815 (3), 1.814	1.805 (1)	1.848	1.841
P1---O1, P2--O1’	2.808, 2.818	2.811	2.747, 2.739	2.800, 2.813
F---C1-5, B---Ct (centroid of Naphthalene)	-	2.968-4.010, 3.565	-	-

Torsion angle	Crystal data		Theor. Data	
	**1**	$\mathbf{1}^{2+}$	**1**	$\mathbf{1}^{2+}$
O1-C6-C1-C2	1.83, 0.05	-7.74	-0.13, 0.12	1.63, 1.27
O2-C7-C4-C3	1.49, -2.93	+3.69	0.11, -0.31	-0.81, 0.51

Table S2: A comparison of the selected bond lengths (Å) and torsional angles (°) of X-ray crystal structures of **2** and **2^{2+}** and the geometry optimized structures of **2** and **2^{2+}**.

Bond	Crystal data		Theor. data	
	**2**	**2^{2+}**	**2**	**2^{2+}**
C1-C2, C1'-C2'	1.444 (4)	1.394 (4)	1.453	1.398
C2-C3, C2'-C3'	1.376 (3)	1.404 (4)	1.382	1.414
C3-C4, C3'-C4'	1.421 (4)	1.376 (4)	1.410	1.380
C4-C5, C4'-C5'	1.412 (4)	1.397 (4)	1.424	1.410
C1-C5', C1'-C5	1.403 (4)	1.412 (4)	1.403	1.419
C5-C5'	1.437 (3)	1.416 (4)	1.437	1.418
C1-C6, C1'-C6'	1.428 (3)	1.483 (4)	1.430	1.491
C4-C7, C4'-C7'	1.437 (4)	1.480 (4)	1.449	1.492
C6-N1, C6'-N1'	1.390 (4)	1.388 (4)	1.391	1.378
C7-N1', C7'-N1	1.415 (5)	1.404 (4)	1.416	1.402
C6-O1, C6'-O1'	1.244 (4)	1.213 (4)	1.251	1.218
C7-O2, C7'-O2'	1.237 (5)	1.208 (4)	1.233	1.210
N1-C8, N1'-C8''	1.454 (3)	1.461(3)	1.465	1.477
C2-P1, C2'-P2'	1.779 (3)	1.824 (3)	1.791	1.867
P1-C32, P1'-C20'	1.807 (3)	1.799 (3)	1.832	1.817
P1-C20, P1'-C26'	1.818 (4)	1.803 (3)	1.843	1.824
P1-C19, P1'-C32'	1.798 (3)	1.798 (3)	1.830	1.815
P1---O1, P2--O1'	2.658	2.738	2.688	2.810
F---C1-5, B---Ct (centroid of Naphthalene)	-	2.968-4.010, 3.565	-	-
**Atoms**	**Crystal data**		**Theor. Data**	
	**2**	**2^{2+}**	**2**	**2^{2+}**
O1-C6-C1-C2	-1.46, 1.46	0.52, -0.52	2.08, -2.09	3.98, -3.98
O2-C7-C4-C3	0.71, -0.71	0.88, -0.88	-1.25, 1.25	-1.28, 1.28

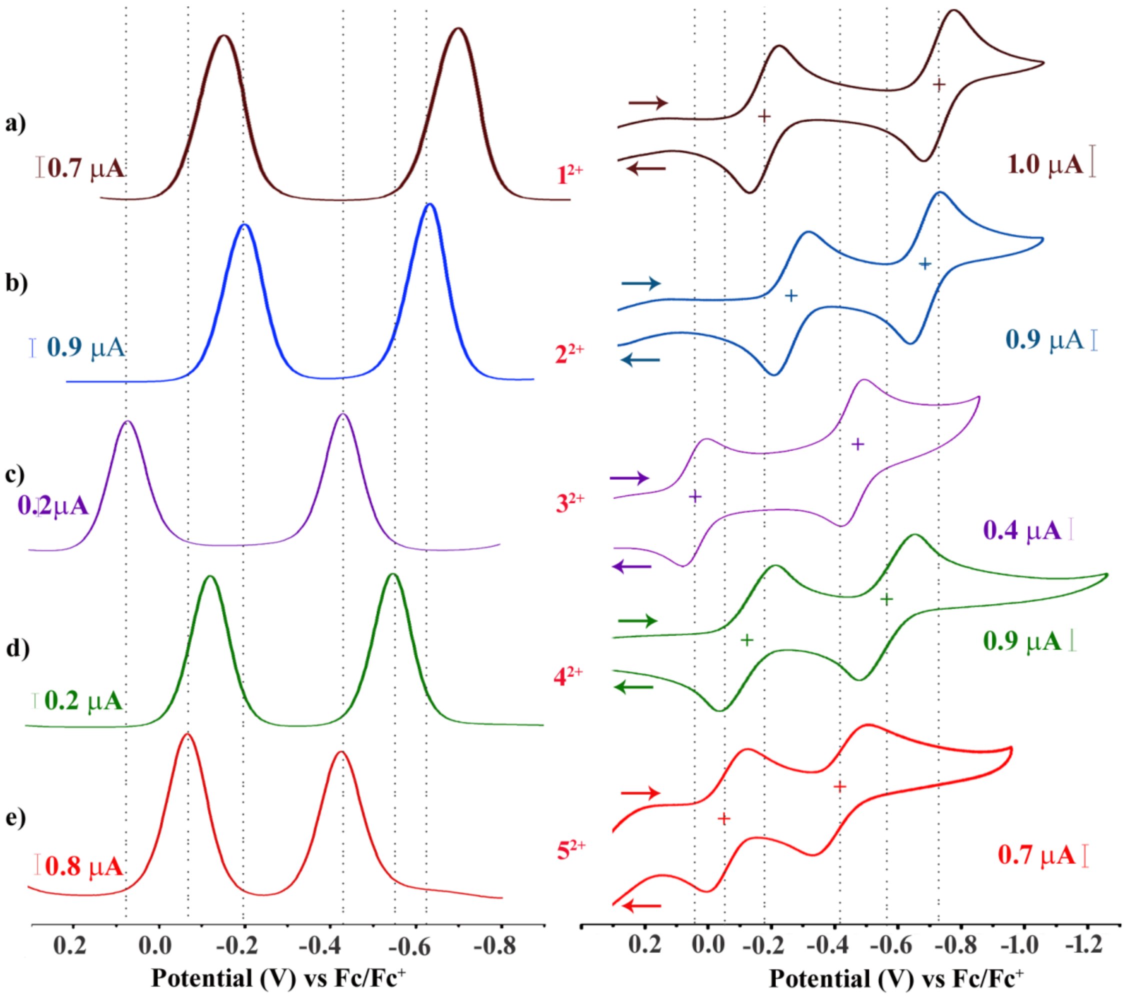

Figure S3: CV and DPV of dicationic compounds a) **1**$^{2+}$, b) **2**$^{2+}$, c) **3**$^{2+}$, d) **4**$^{2+}$ and e) **5**$^{2+}$. Conditions: 5×10^{-4} M in DCM; reference electrode, Ag/AgCl; working and auxiliary electrodes, Pt with 0.1 M n-Bu_4NPF_6 and (Fc/Fc^+); 298 K; scan rate, 200 mV/s.

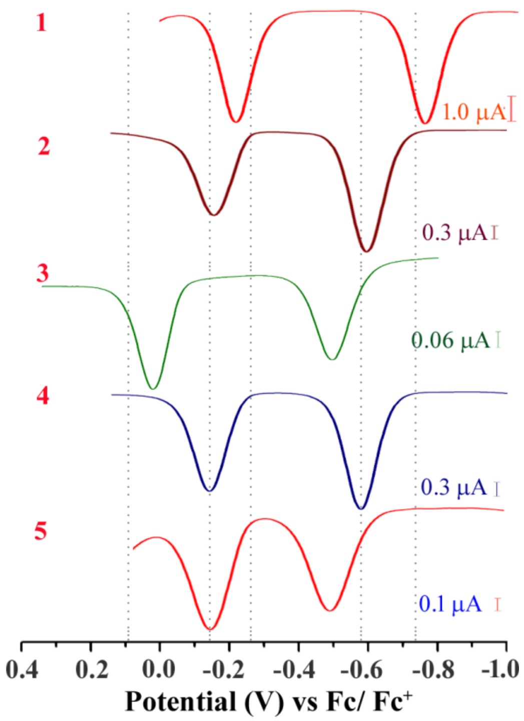

Figure S4: DPV of direduced molecules **1**, **2**, **3**, **4** and **5**. Conditions: 5 × 10^{-4} M in DCM; reference electrode, Ag/AgCl; working and auxiliary electrodes, Pt with 0.1 M Bu_4NPF_6 and (Fc/Fc^+); 298 K; scan rate, 200 mV/s.

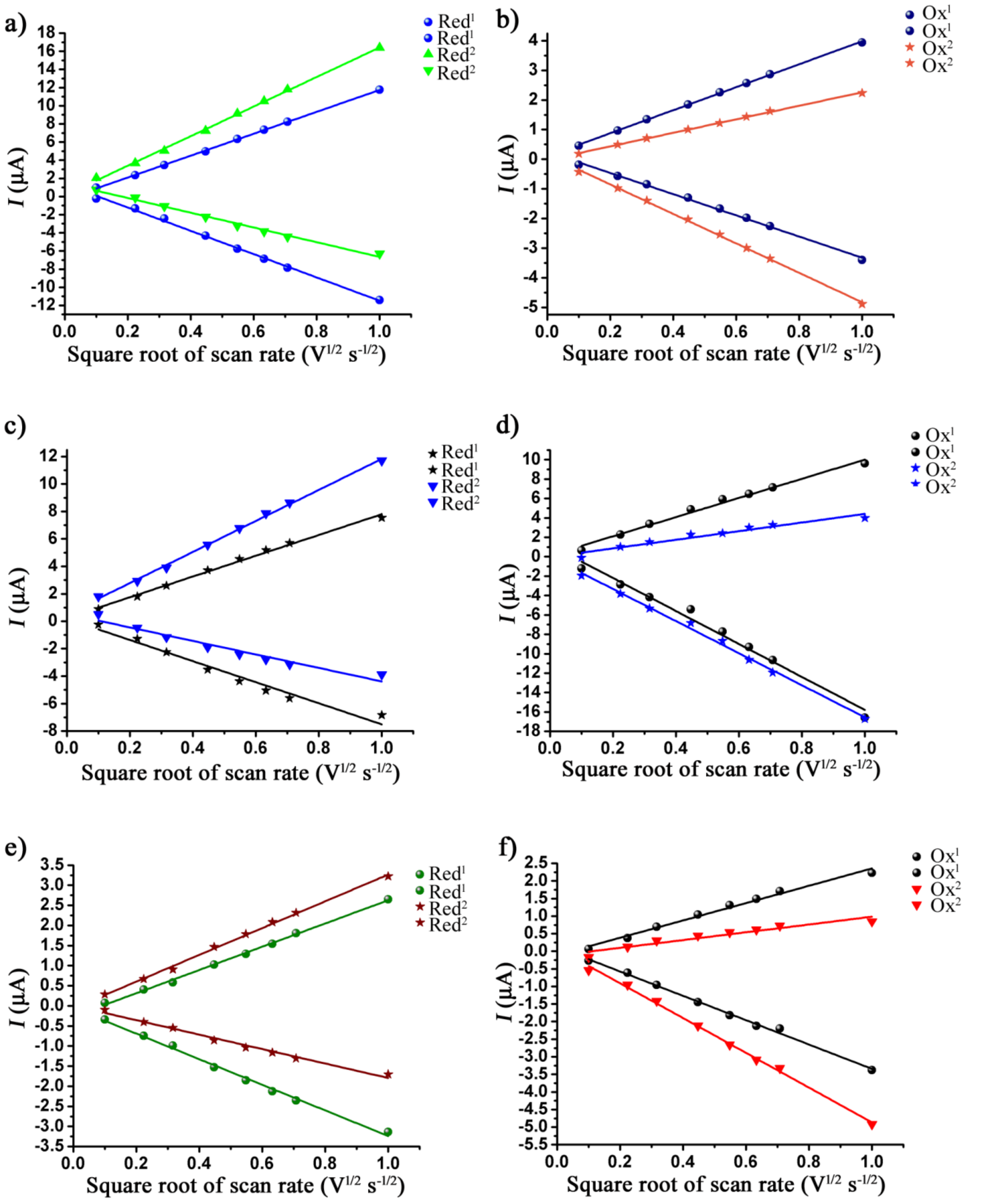

Figure S5: Square root of scan rate vs current peak of the redox wave (anodic and cathodic) for a) **1**$^{2+}$, c) **2**$^{2+}$and e) **5**$^{2+}$dicationic molecules and b) **1**, d) **2** and f) **5** direduced molecules.

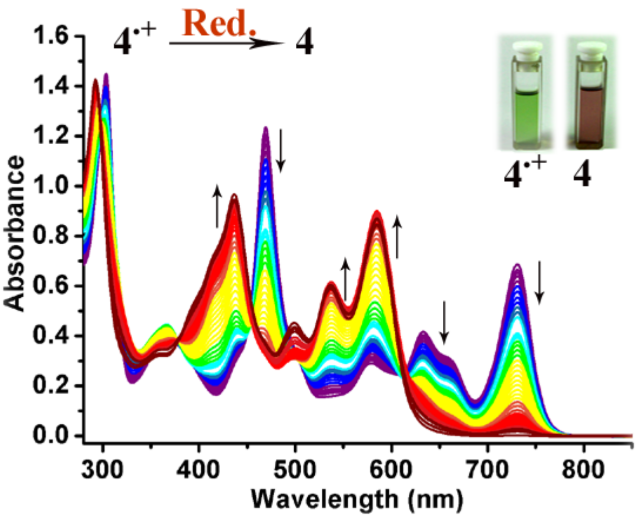

Figure S6: Spectra showing the gradual transformation of **4**$^{\bullet+}$ to **4** due to CN^- induced electron transfer in DMF (5 x 10^{-5} M).

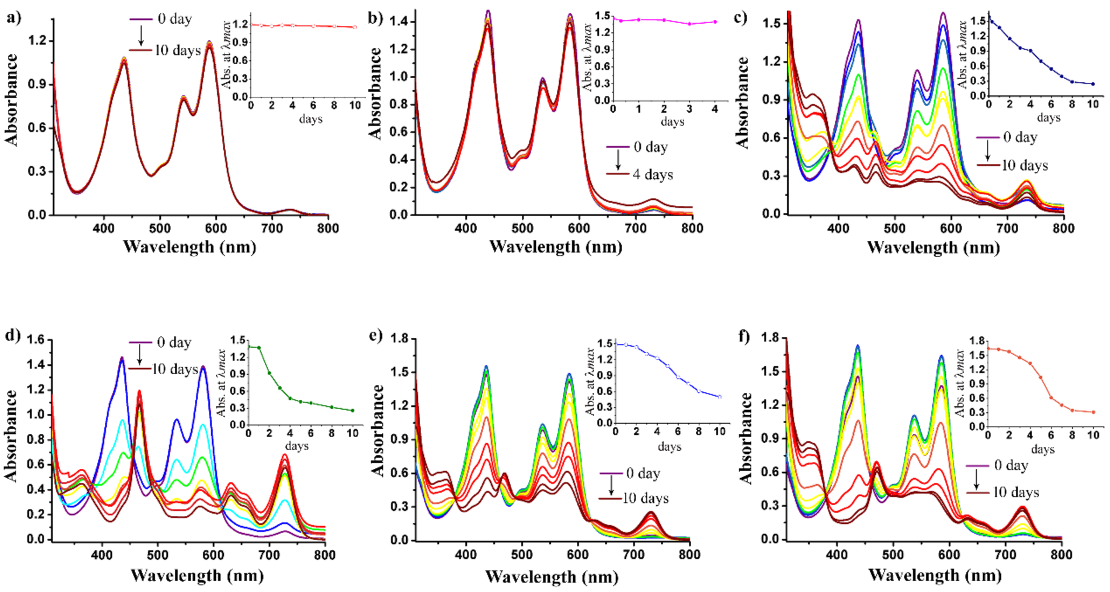

Figure S7: UV-Vis spectra showing the stability of **4** in a) toluene; b) DCM; c) THF; d) MeCN; e) DMF and f) DMSO at 5 x 10^{-5} M. [All the solutions were prepared under ambient conditions].

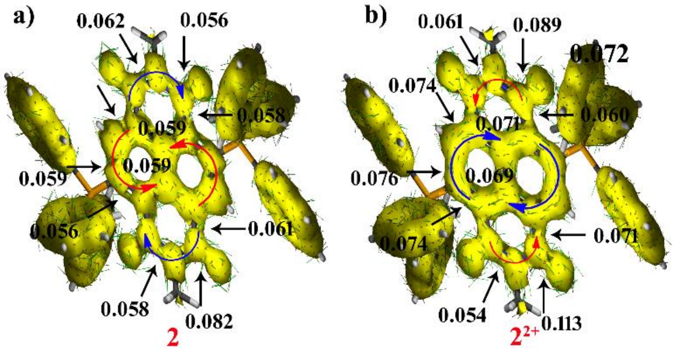

Figure S8: AICD isosurface plots of a) **2**, and b) $\mathbf{2^{2+}}$. The induced ring current vectors are plotted on the AICD isosurface to designate the diatropic and paratropic ring currents. The AICD plots are plotted at 0.05 isosurface value.

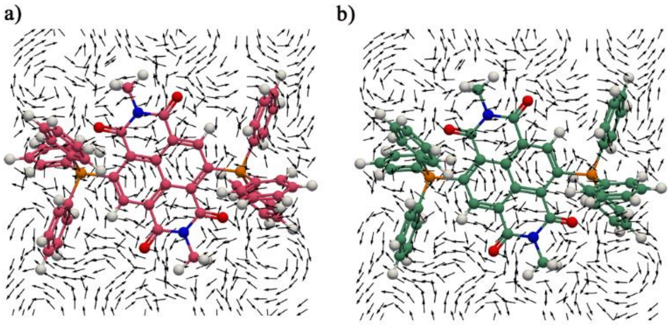

Figure S9: The magnetically induced current density of a) **2**, and b) $\mathbf{2^{2+}}$ calculated in a plane placed at 1.0 Å above the molecular plane. Diatropic currents are assumed to circle clockwise and the paratropic ones circle anticlockwise.

Table S3: The diatropic, paratropic and net current strengths (current strength susceptibility in nA T^{-1}) calculated at the B3LYP/6311++G(d,p) level. The numbering of the molecules is given in figure.

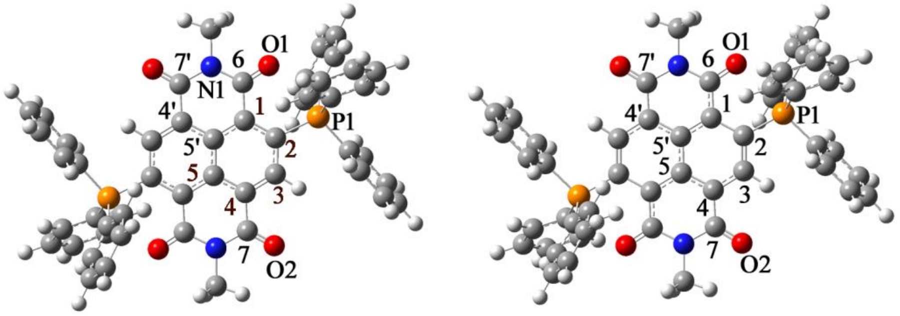

Molecule 2 and 2^{2+}						
**Bond**	**Diatropic**		**Paratropic**		**Net Current**	
	**2**	**2^{2+}**	**2**	**2^{2+}**	**2**	**2^{2+}**
C1-C2	8.43	17.33	-12.38	-8.82	-3.95	8.50
C2-C3	8.72	17.43	-16.88	-12.36	-8.15	5.07
C3-C4	7.77	17.18	-11.02	-7.42	-3.25	9.75
C4-C5	9.96	17.76	-16.41	-11.51	-6.45	6.24
C1-C5’	8.01	16.69	-17.02	-12.10	-9.00	4.58
C5-C5’	6.96	13.00	-16.38	-13.60	-9.42	-0.59
C1-C6	11.11	10.52	-13.36	-15.83	-2.25	-5.30
C4’-C7’	9.28	7.27	-4.52	-6.63	4.75	0.63
C6-N1	10.55	7.60	-5.98	-7.30	4.57	0.29
C7’-N1	9.65	6.99	-5.64	-7.07	4.00	-0.08
C6-O1	11.74	12.09	-18.31	-18.41	-6.56	-6.32
C7-O2	11.04	12.09	-16.36	-16.95	-5.32	-4.85
C2-P1	8.90	9.71	-7.07	-7.50	1.82	2.21

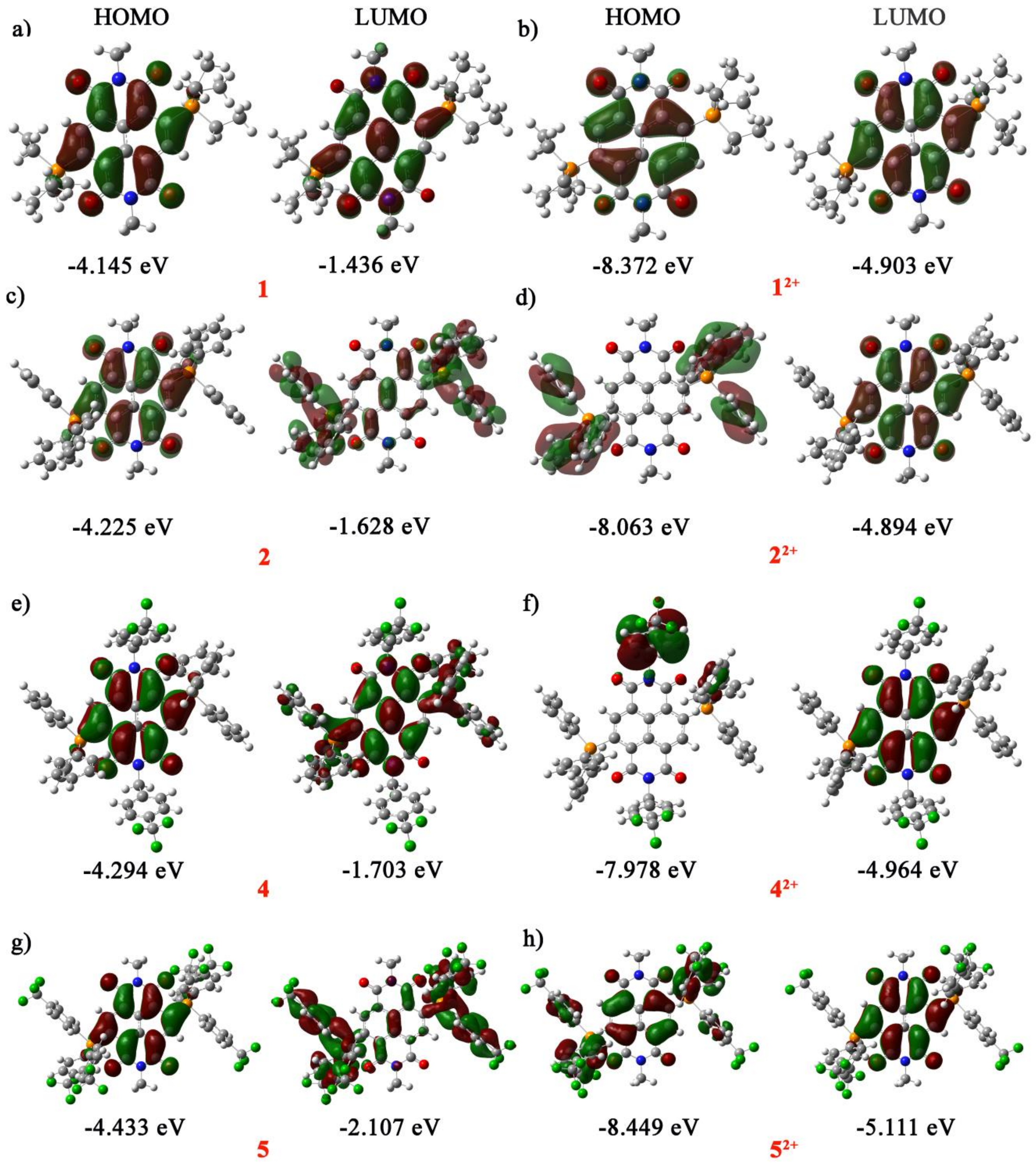

Figure S10: Frontier molecular orbital diagrams and the corresponding energy values of a) **1**, b) **1**$^{2+}$, c) **2**, d) **2**$^{2+}$, e) **4**, f) **4**$^{2+}$, g) **5**, h) **5**$^{2+}$ at 6311++G(d,p) for **1-4** and 631+G(d,p) basis set for **5** with IEFPCM model using DCM as solvent.

Table S4: Natural population analysis of molecule **1**, $\mathbf{1}^{2+}$, **2** and $\mathbf{2}^{2+}$.

Atom	Natural Population Analysis			
	**1**	$\mathbf{1}^{2+}$	**2**	$\mathbf{2}^{2+}$
C1, C1’	-0.172, -0.204	-0.031, -0.070	-0.099	-0.023
C2, C2’	-0.445, -0.435	-0.301, -0.284	-0.379	-0.301
C3, C3’	-0.130, -0.129	-0.130, -0.126	-0.149	-0.131
C4, C4’	-0.209, -0.249	-0.067, -0.117	-0.220	-0.078
C5, C5’	0.004, 0.143	-0.043, 0.099	-0.014	-0.018
C6, C6’	0.641, 0.634	0.689, 0.682	0.602	0.668
C7, C7’	0.651	0.683	0.655	0.692
N1, N1’	-0.483, -0.480	-0.479, -0.483	-0.477	-0.486
O1, O1’	-0.753, -0.744	-0.608, -0.601	-0.704	-0.568
O2, O2’	-0.684	-0.531, -0.533	-0.671	-0.542
P1, P1’	1.594, 1.608	1.559, 1.567	1.624	1.631

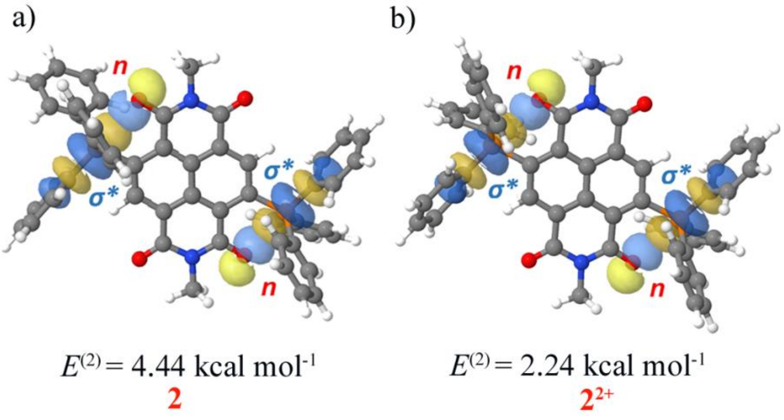

Figure S11: NBO diagram of a) **2** and b) $\mathbf{2}^{2+}$ depicting intramolecular $n_O \rightarrow \sigma_{P-C^*}$ orbital interaction between the donor O lone pair of the imide groups in $\mathbf{2}^{+}$ and $\mathbf{2}^{2+}$ and the acceptor P−C σ* antibonding orbitals.

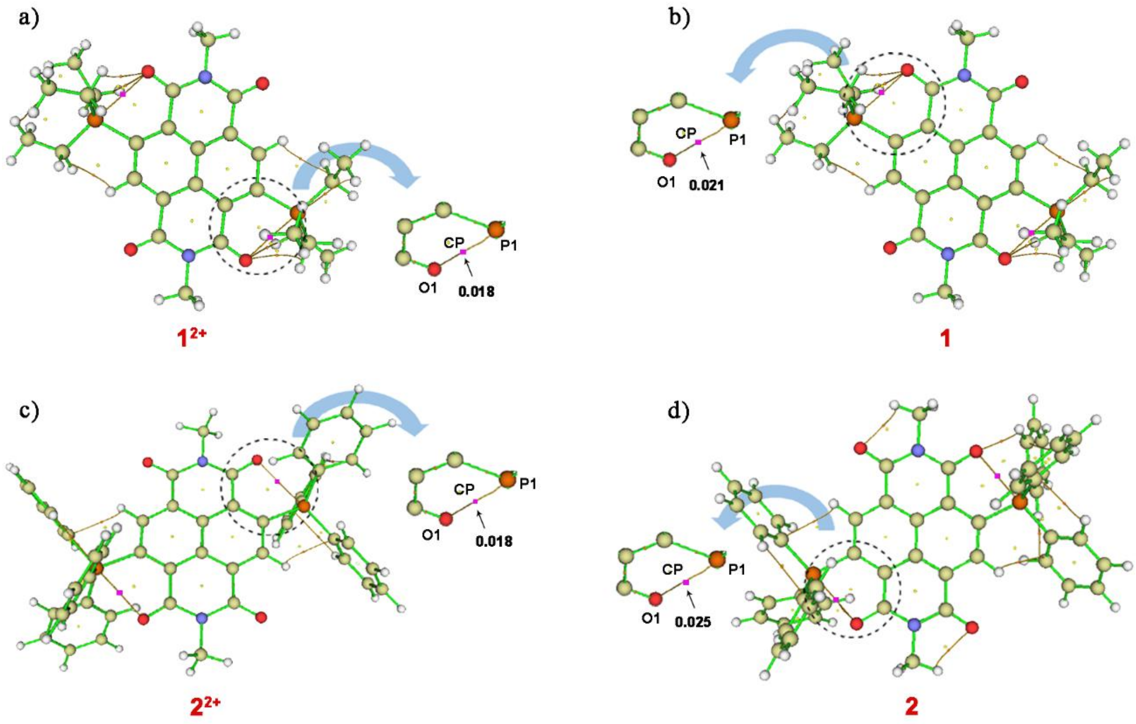

Figure S12: 3D bond critical paths of a) $\mathbf{1}^{2+}$, b) **1**, c) $\mathbf{2}^{2+}$ and d) **2** are represented and the non-bonding P---O interactions are highlighted with dashed circles. The electron density, $\rho(\mathbf{r}_b)$ values of the P---O interactions have been specified.

Table S5: Crystallographic data for molecule **1**, **1^{2+}**, **2**, and **2^{2+}**.

	**1**	**1^{2+}**	**2**	**2^{2+}**
Empirical formula	$C_{40}H_{46}N_2O_4P_2$	$C_{40}H_{46}B_2F_8N_2O_4P_2$	$C_{74}H_{66}N_2O_4P_2$	$C_{74}H_{66}B_2F_8N_2O_4P_2$
Formula weight	680.29	854.35	1108.45	1282.45
Temperature/K	100(2)	100(2)	100(2)	100(2)
Crystal system	monoclinic	triclinic	triclinic	monoclinic
Space group	C2	P-1	P-1	$P2_1/n$
a/Å	18.1243(13)	9.2335(6)	11.0052(6)	11.567(16)
b/Å	12.7829(10)	10.3160(7)	13.5844(7)	17.559(3)
c/Å	19.6169(19)	11.4148(8)	14.2986(8)	15.777(2)
α/°	90	94.362(3)	103.374(2)	90
β/°	117.061(2)	91.419(2)	94.280(3)	98.559(4)
γ/°	90	116.416(3)	111.830(2)	90
Volume/Å^3	4047.3(6)	968.83(12)	1899.74(18)	3168.6(8)
Z	4	1	1	2
ρ_{calc}g/cm^3	1.117	1.464	1.186	1.345
μ/mm^{-1}	0.146	0.196	0.247	0.146
F(000)	1448.0	444.0	712.0	1336.0
2Θ range for data collection/°	2.247 to 28.33	2.21 to 28.27	2.29 to 28.31	2.32 to 28.20
Reflections collected	44984	30338	77158	64232
Independent reflections	10081	4823	9511	7921
Goodness-of-fit on F^2	1.342	1.100	1.393	1.209
R1 [I>=2σ (I)]	0.0312	0.0467	0.0813	0.0835
wR2(reflection)	0.0861	0.1423	0.2181	0.1996

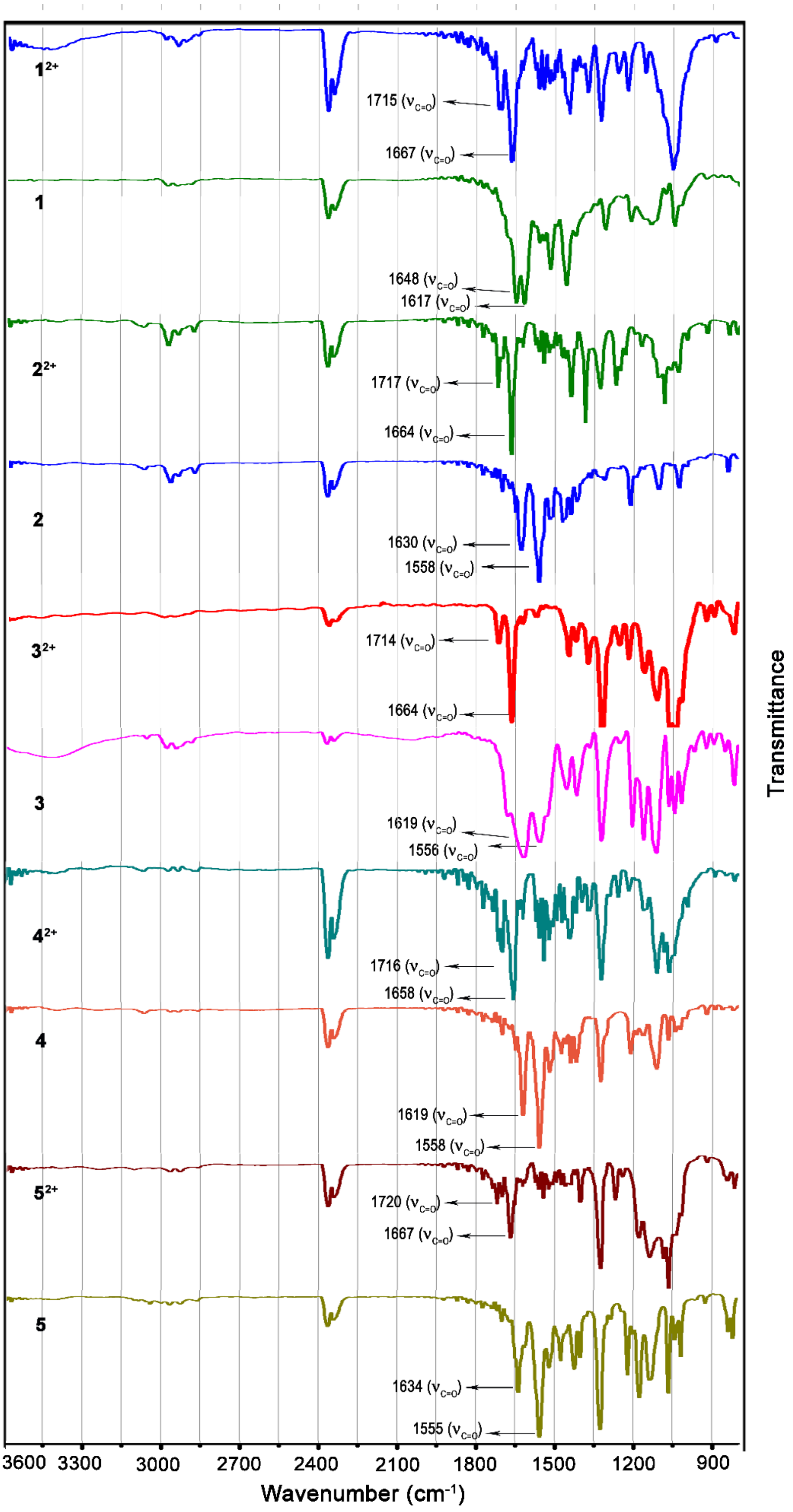

Figure S13: FT-IR spectra of compounds **1-5** and their dicationic $\mathbf{1}^{2+}$-$\mathbf{5}^{2+}$ states in KBr pellet.

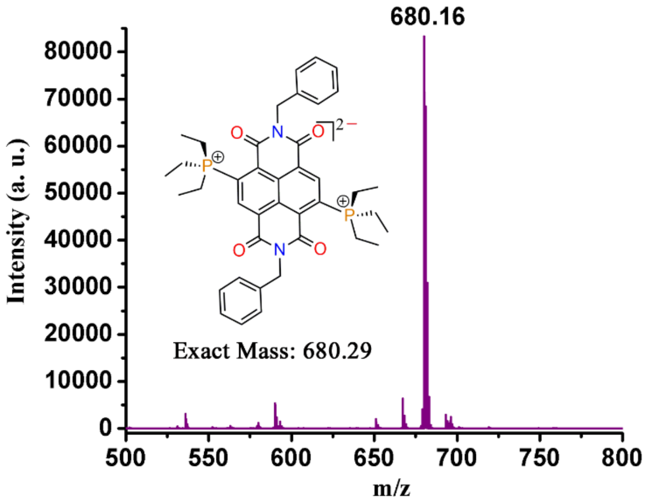

Figure S14: MALDI-TOF mass spectrometry of molecule **1**.

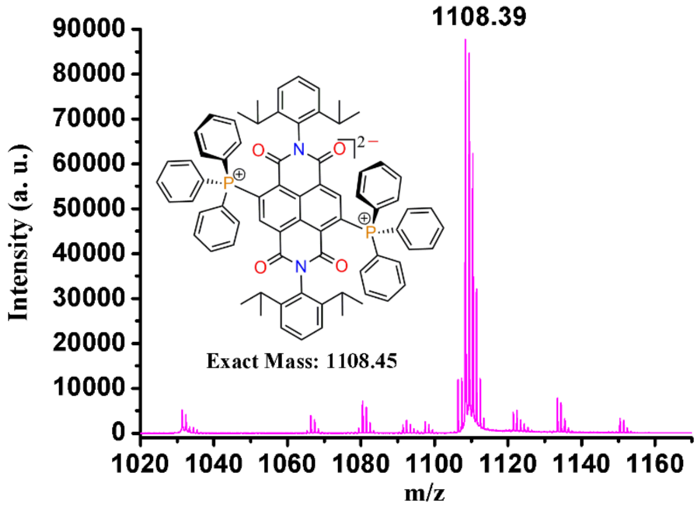

Figure S15: MALDI-TOF mass spectrometry of molecule **2**.

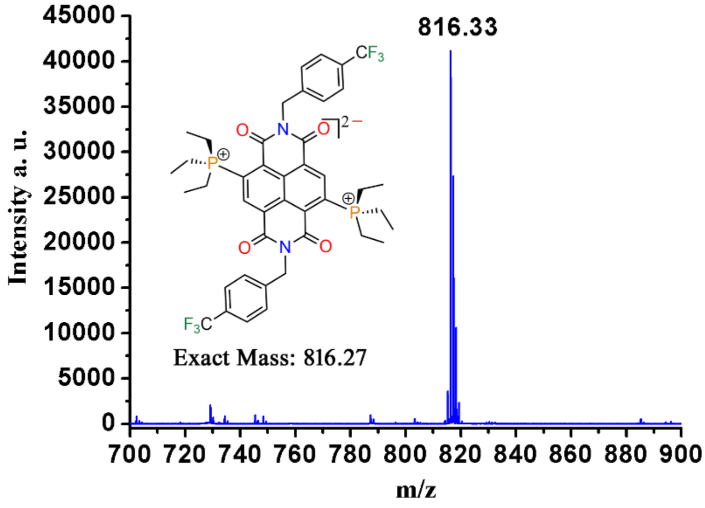

Figure S16: MALDI-TOF mass spectrometry of molecule **3**.

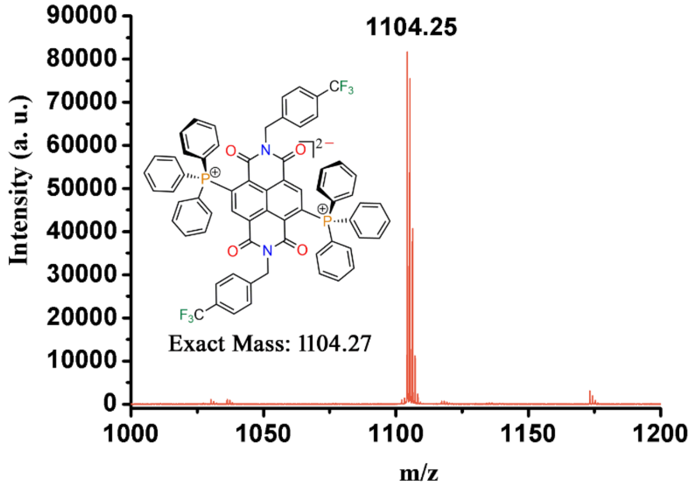

Figure S17: MALDI-TOF mass spectrometry of molecule **4**.

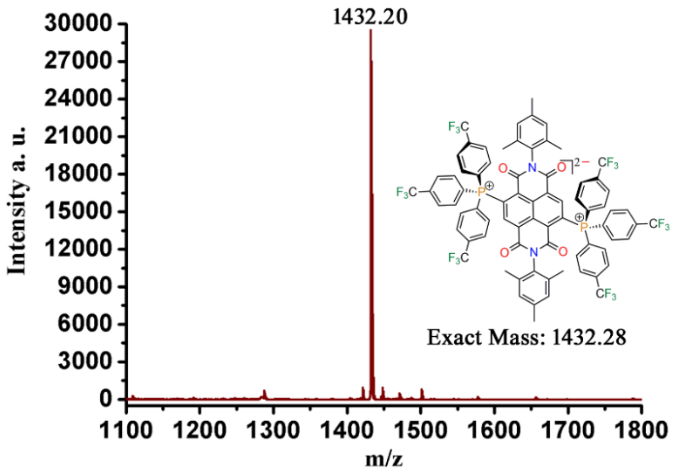

Figure S18: MALDI-TOF mass spectrometry of molecule **5**.

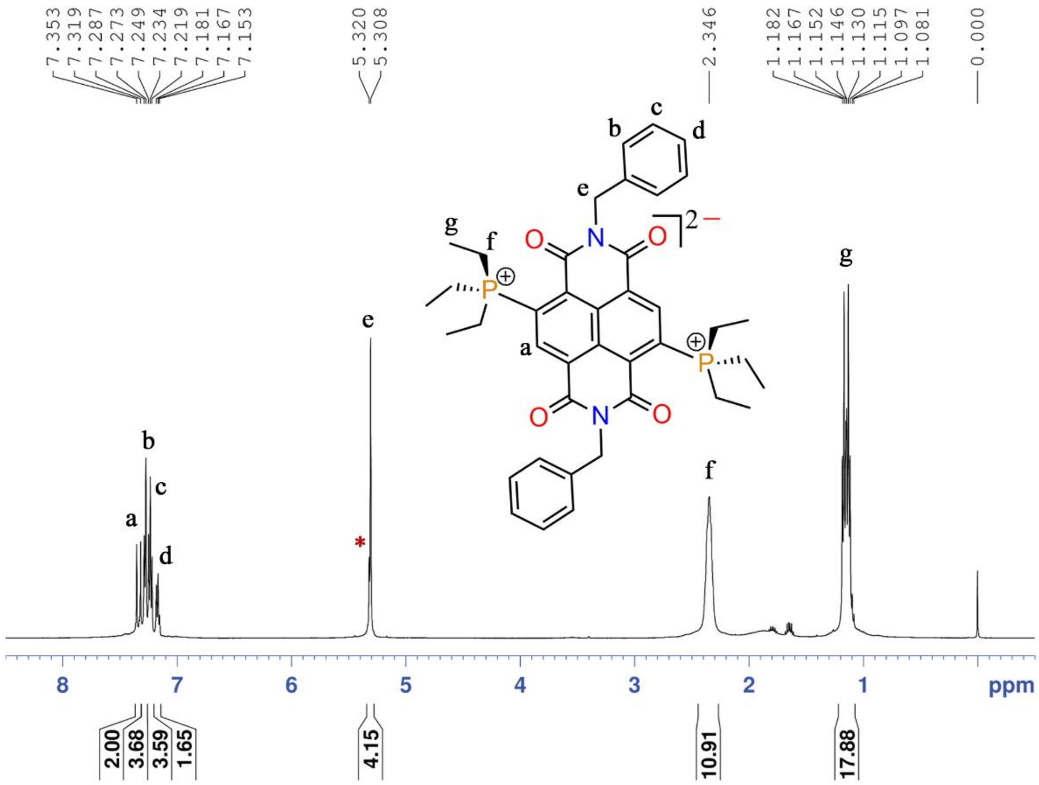

Figure S19: 500 MHz ^{1}H NMR spectrum of molecule **1** at RT in CD_2Cl_2.

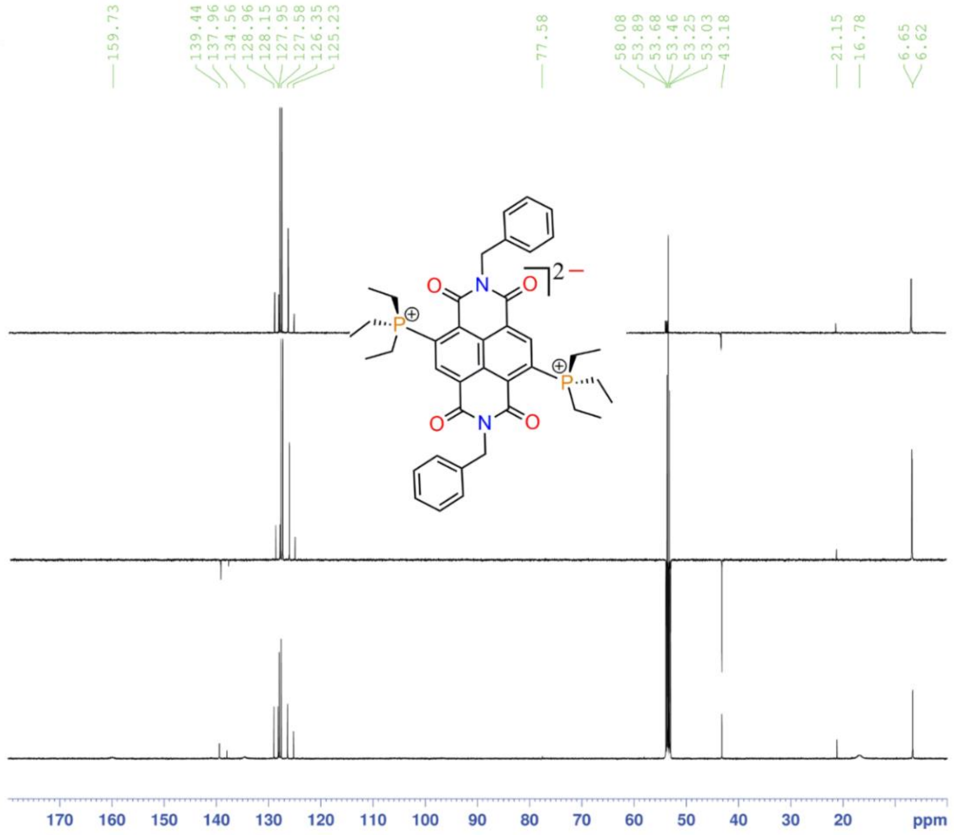

Figure S20: 125 MHz ^{13}C NMR, APT and DEPT-135 spectra of molecule **1** at RT in CD_2Cl_2.

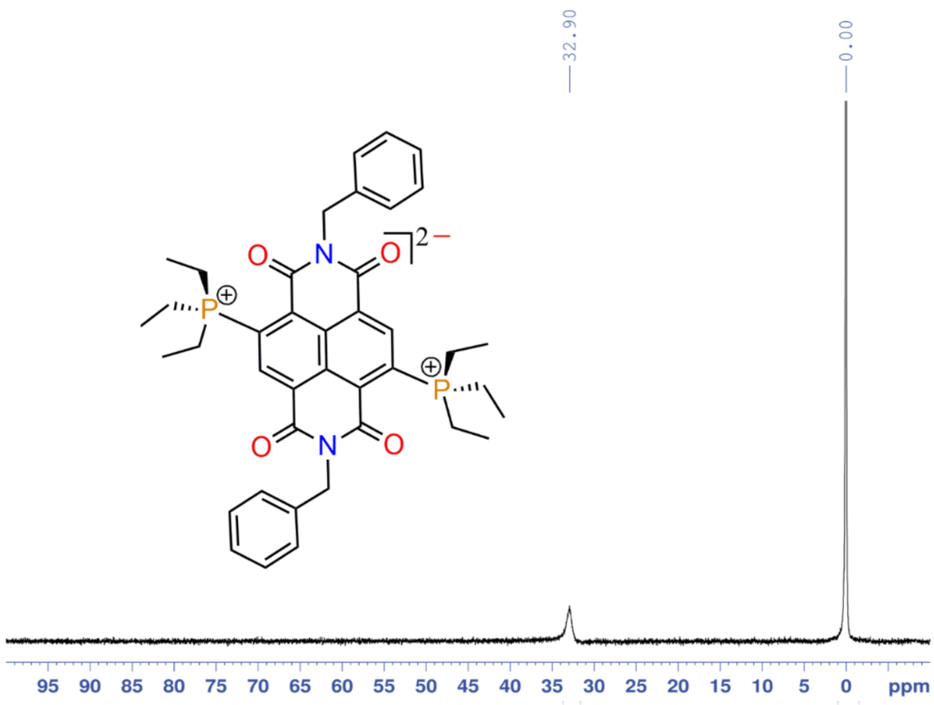

Figure S21: 202 MHz ^{13}P NMR spectrum of molecule **1** at RT in CD_2Cl_2.

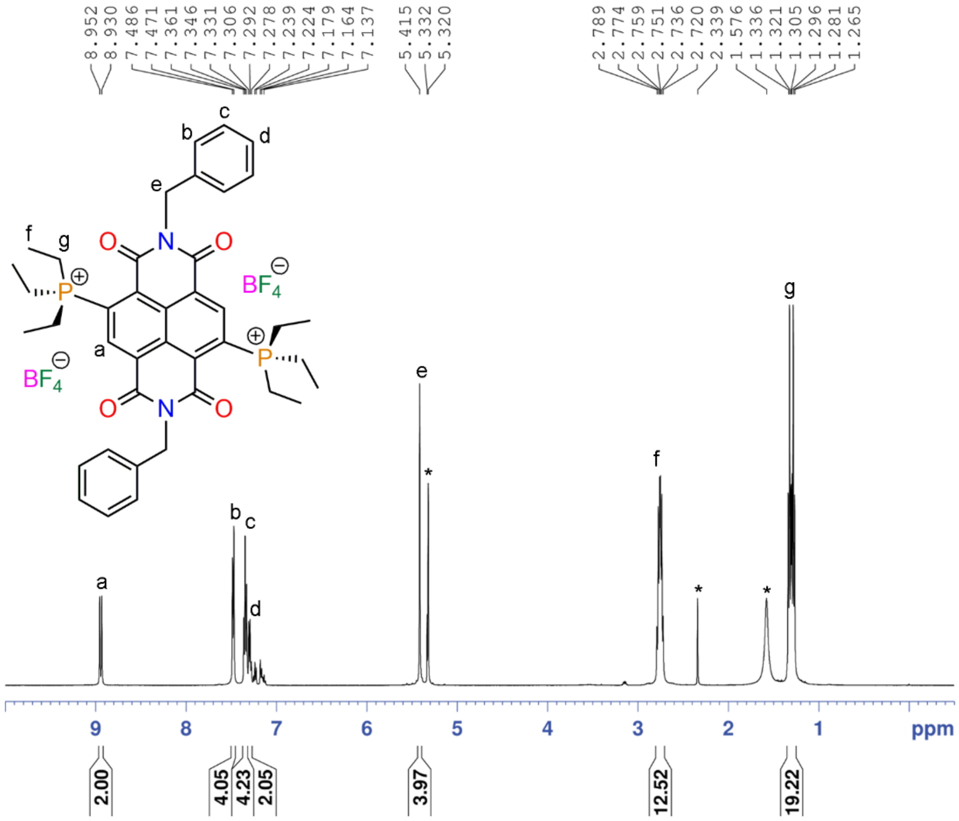

Figure S22: 500 MHz ^{1}H NMR spectrum of molecule **1^{2+}.2BF$_4^{2-}$** at RT in CD_2Cl_2.

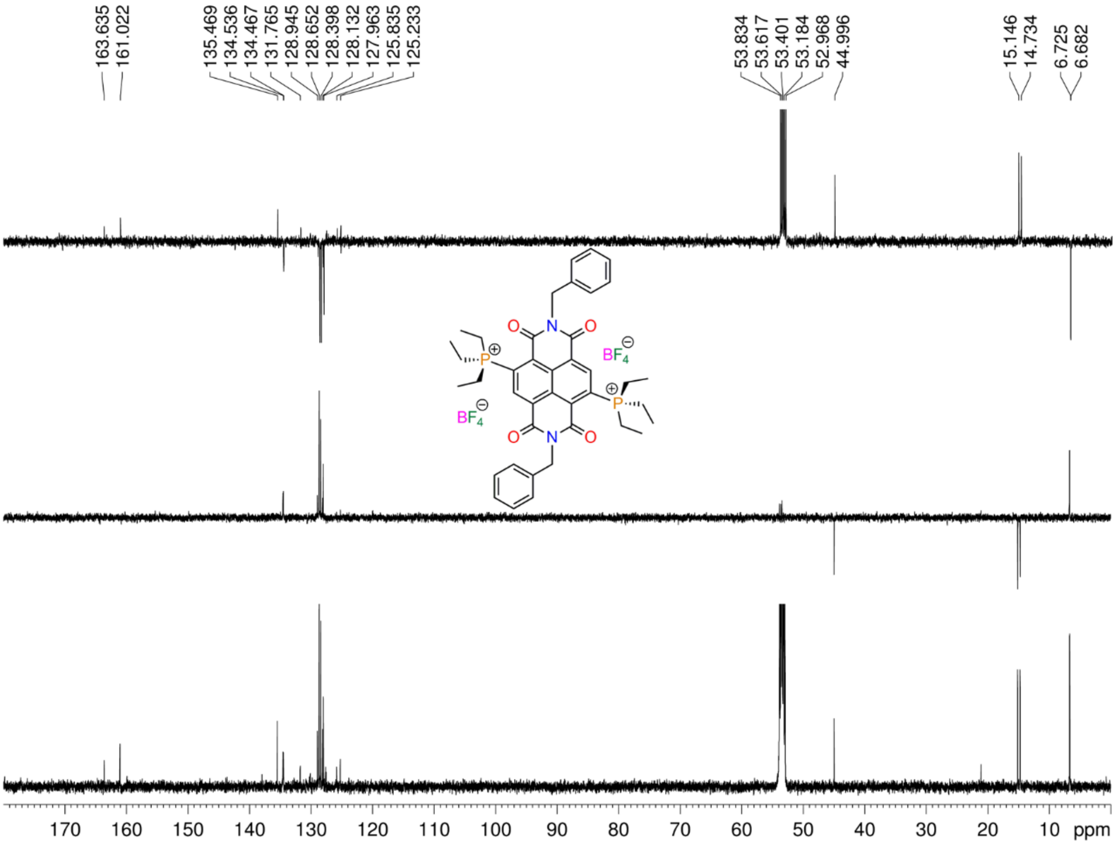

Figure S23: 125 MHz ^{13}C NMR, APT and DEPT-135 spectra of molecule **1^{2+}.2BF$_4$$^{2-}$** at RT in CD_2Cl_2.

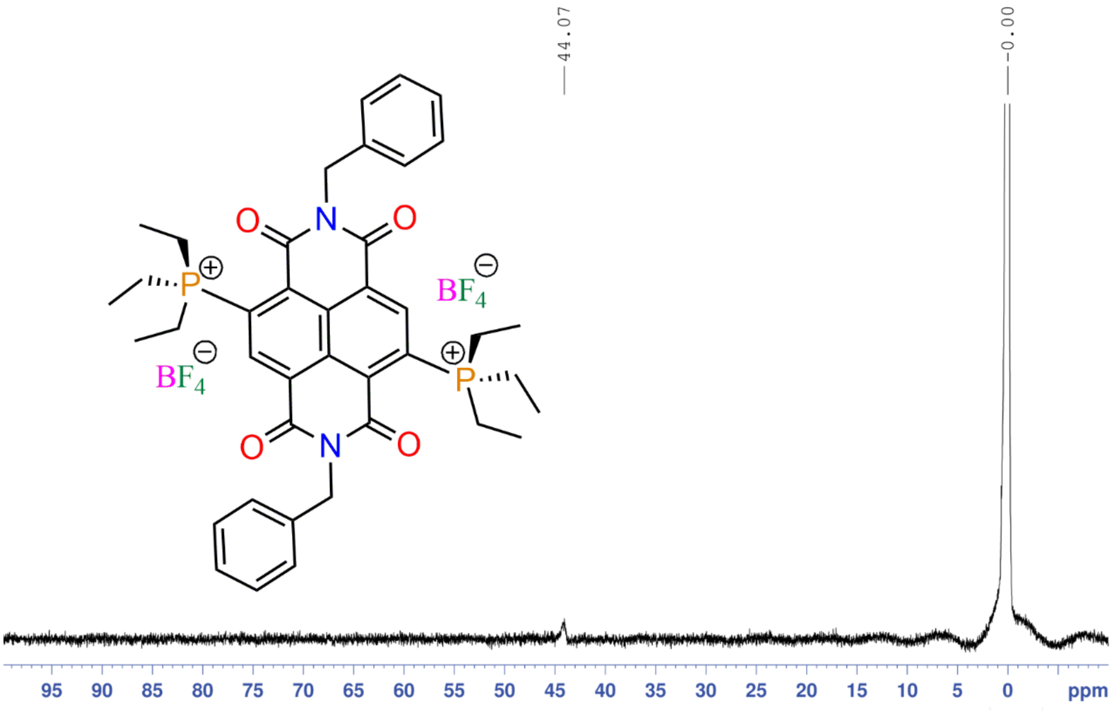

Figure S24: 202 MHz ^{13}P NMR spectrum of molecule **1^{2+}.2BF$_4$$^{2-}$** at RT in CD_2Cl_2.

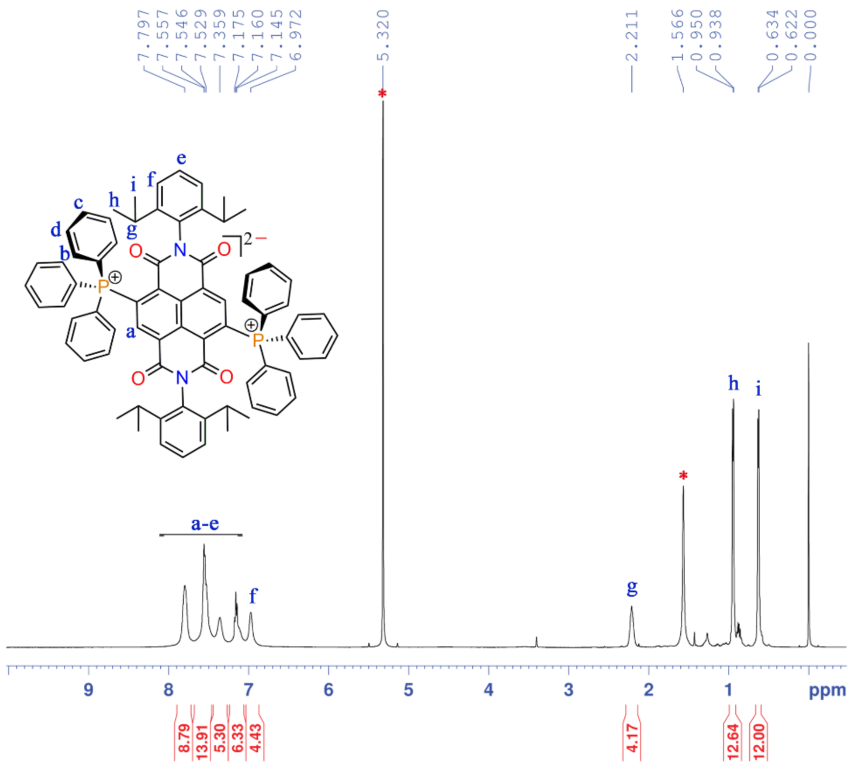

Figure S25: 500 MHz ^1H NMR spectrum of molecule **2** at RT in CD_2Cl_2.

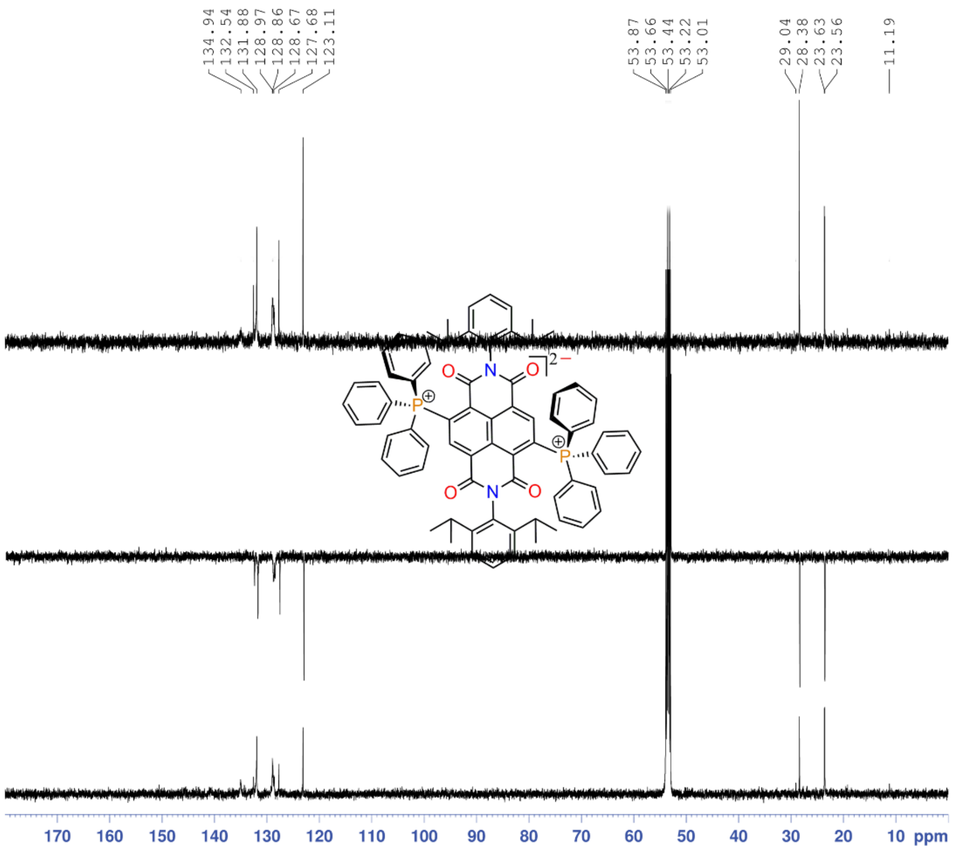

Figure S26: 125 MHz ^{13}C NMR, APT and DEPT-135 spectra of molecule **2** at RT in CD_2Cl_2.

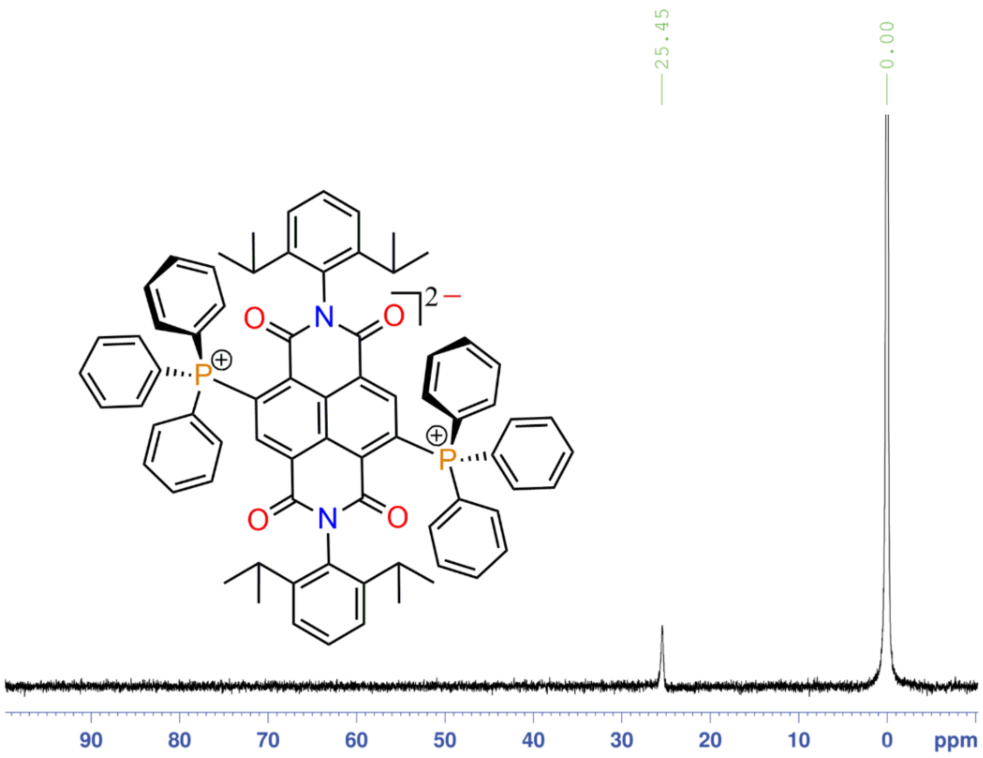

Figure S27: 202 MHz ^{13}P NMR spectrum of molecule **2** at RT in CD_2Cl_2.

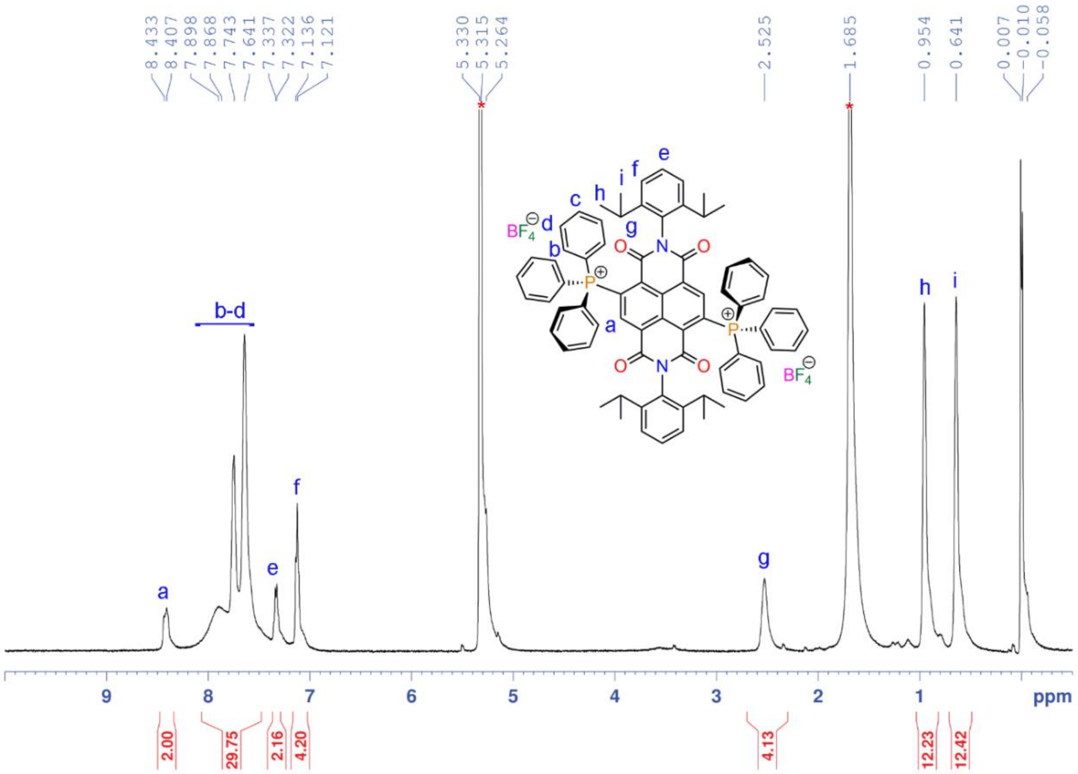

Figure S28: 500 MHz ^{1}H NMR spectrum of molecule **2^{2+}.2BF$_4^{2-}$** at RT in CD_2Cl_2.

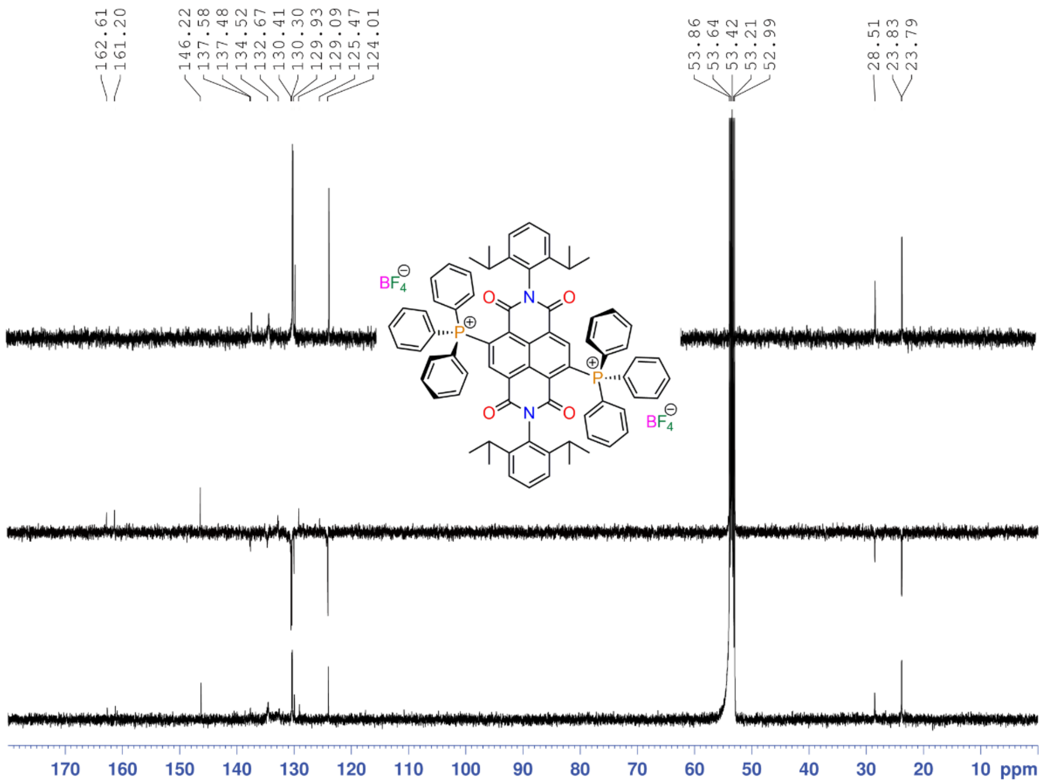

Figure S29: 125 MHz ^{13}C NMR, APT and DEPT-135 spectra of molecule **2^{2+}.2BF$_4^{2-}$** at RT in CD_2Cl_2.

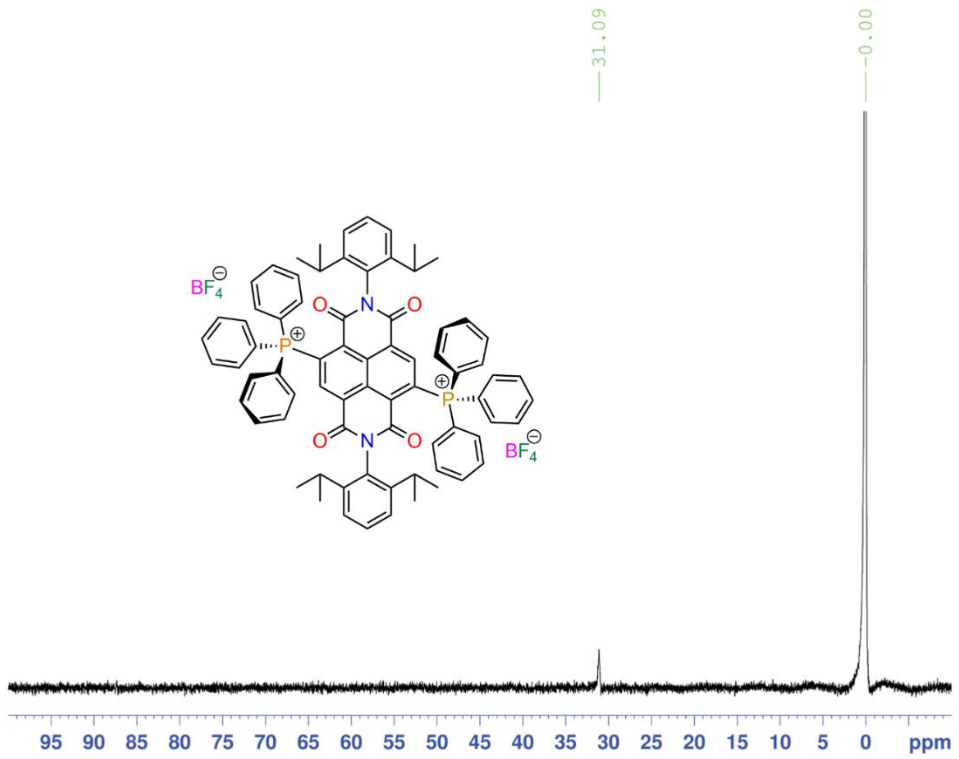

Figure S30: 202 MHz ^{13}P NMR spectrum of molecule **2^{2+}.2BF$_4^{2-}$** at RT in CD_2Cl_2.

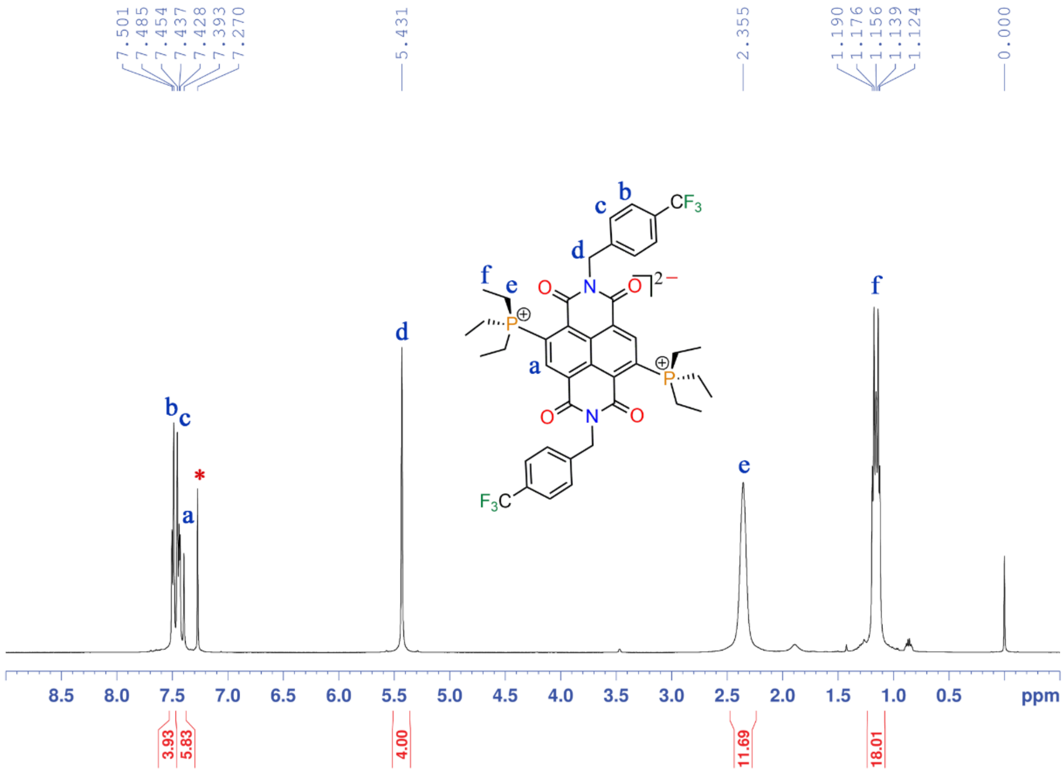

Figure S31: 500 MHz ^{1}H NMR spectrum of molecule **3** at RT in $CDCl_3$.

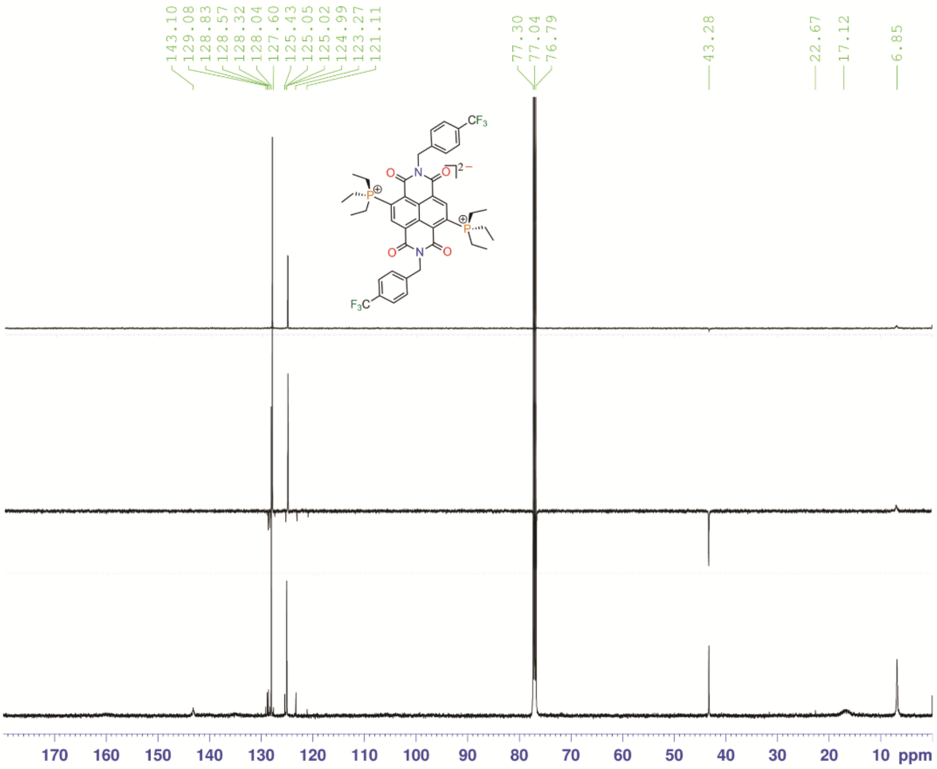

Figure S32: 125 MHz ^{13}C NMR, APT and DEPT-135 spectra of molecule **3** at RT in $CDCl_3$.

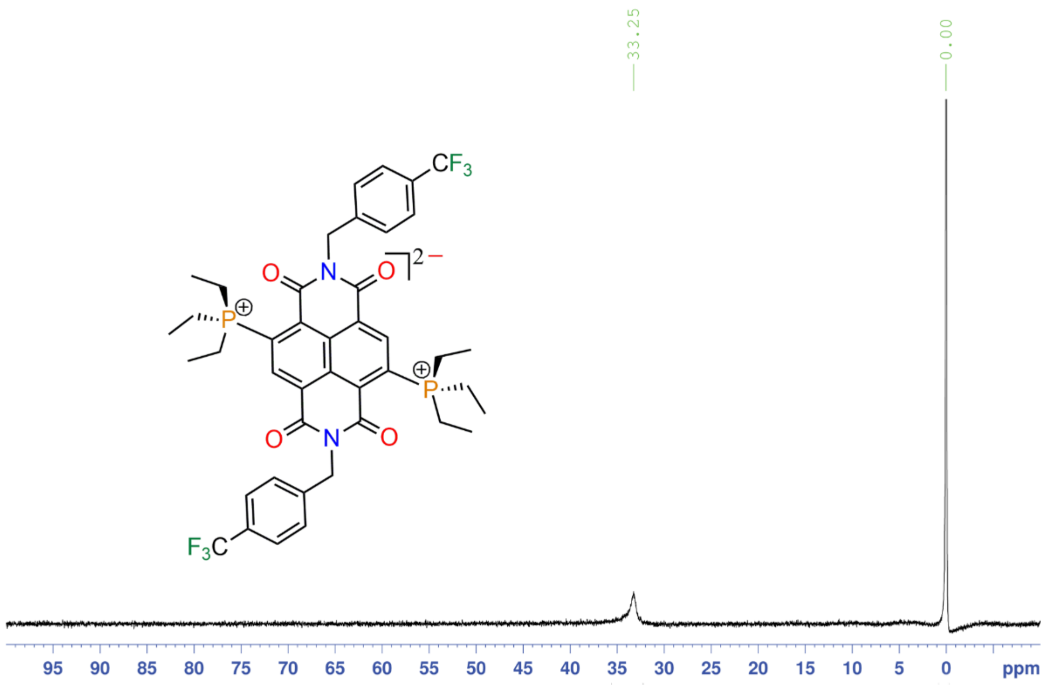

Figure S33: 202 MHz ^{13}P NMR spectrum of molecule **3** at RT in $CDCl_3$.

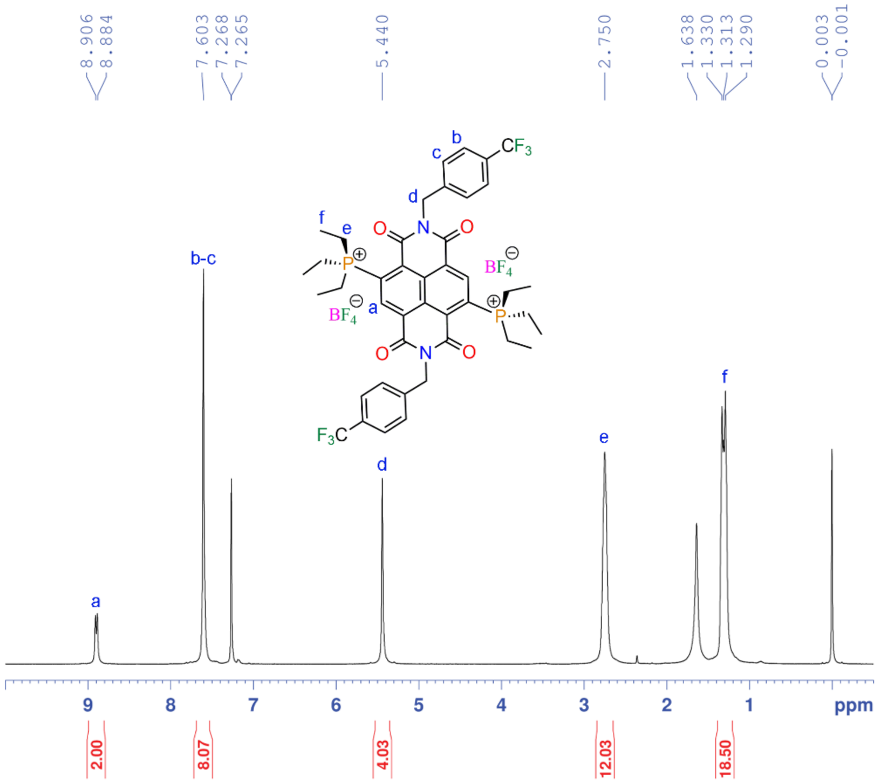

Figure S34: 500 MHz ^{1}H NMR spectrum of molecule $\mathbf{3^{2+}.2BF_4^{2-}}$ at RT in $CDCl_3$.

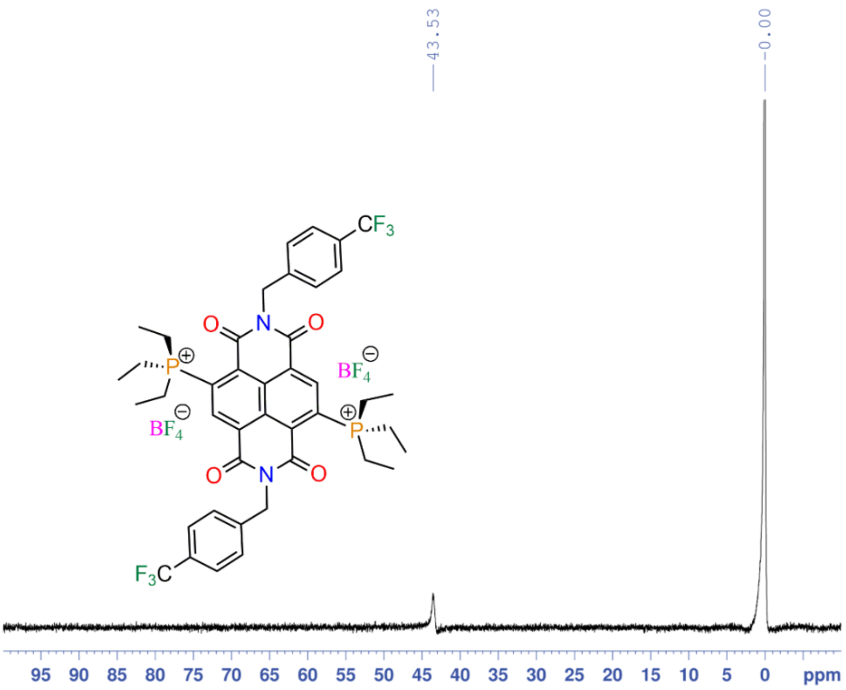

Figure S35: 202 MHz ^{13}P NMR spectrum of molecule $\mathbf{3^{2+}.2BF_4^{2-}}$ at RT in $CDCl_3$.

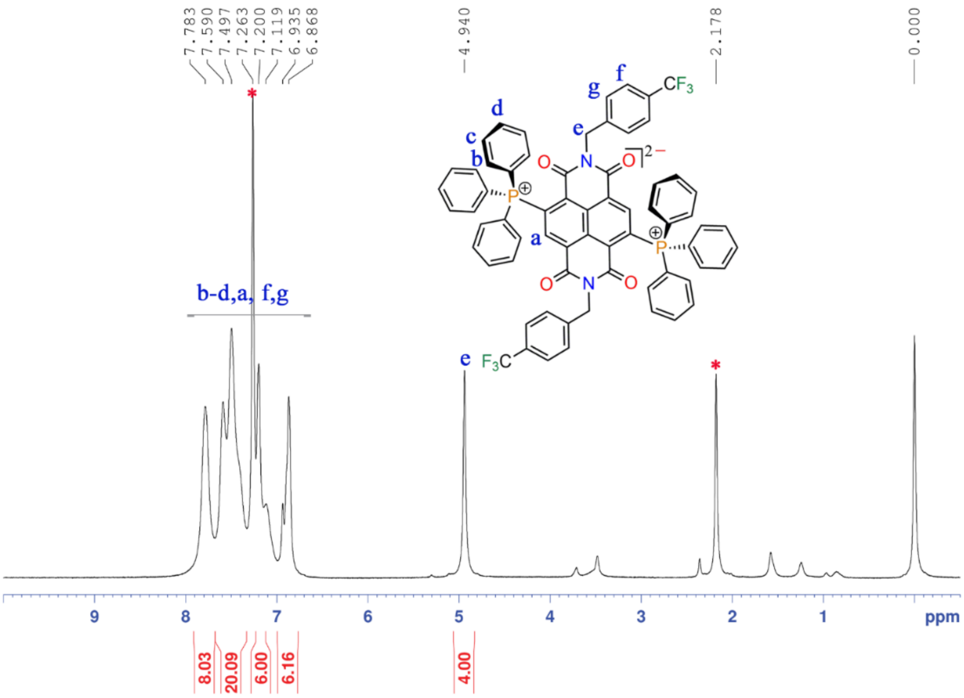

Figure S36: 500 MHz ^{1}H NMR spectrum of molecule **4** at RT in $CDCl_3$.

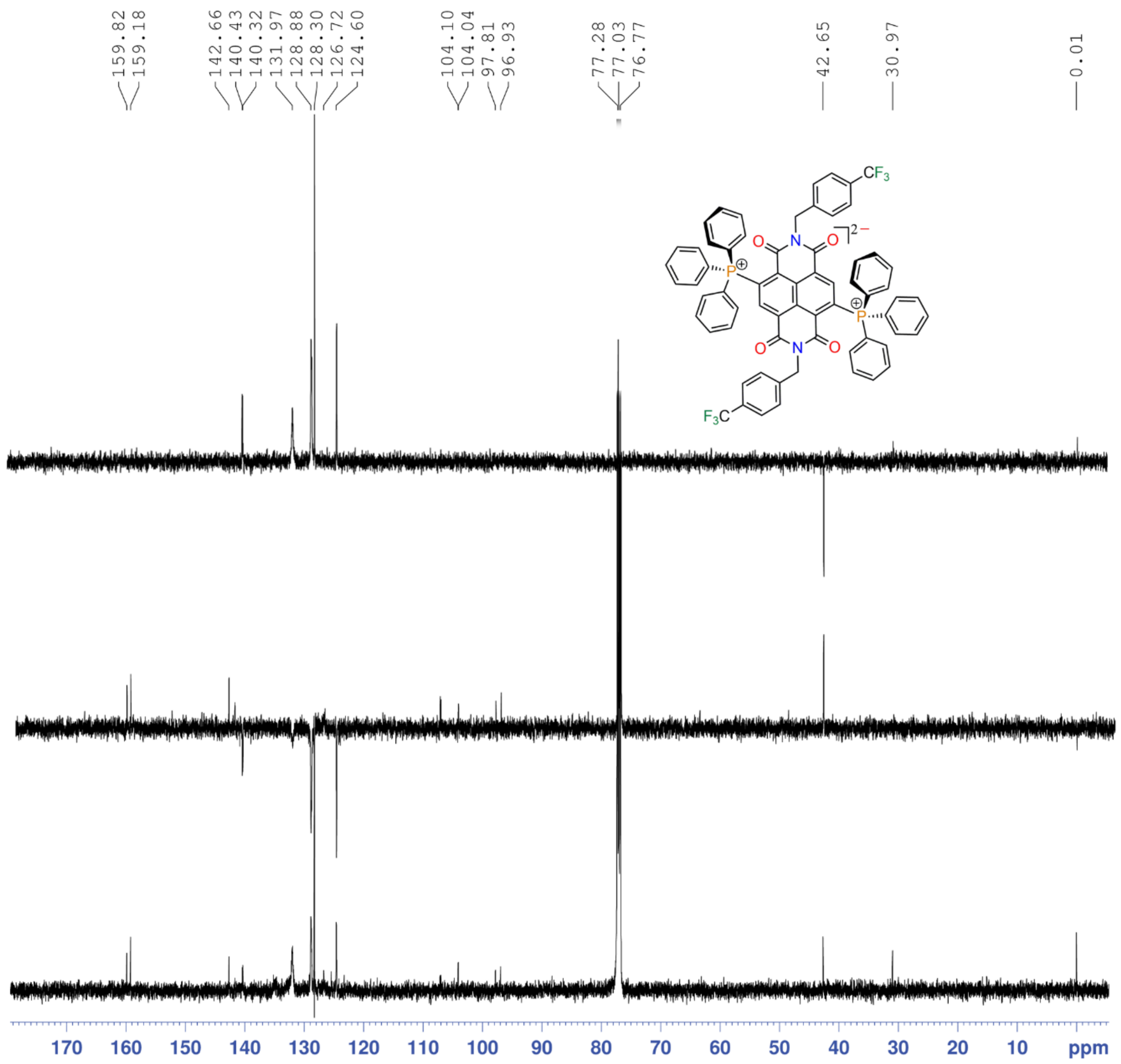

Figure S37: 125 MHz ^{13}C NMR, APT and DEPT-135 spectra of molecule **4** at RT in $CDCl_3$.

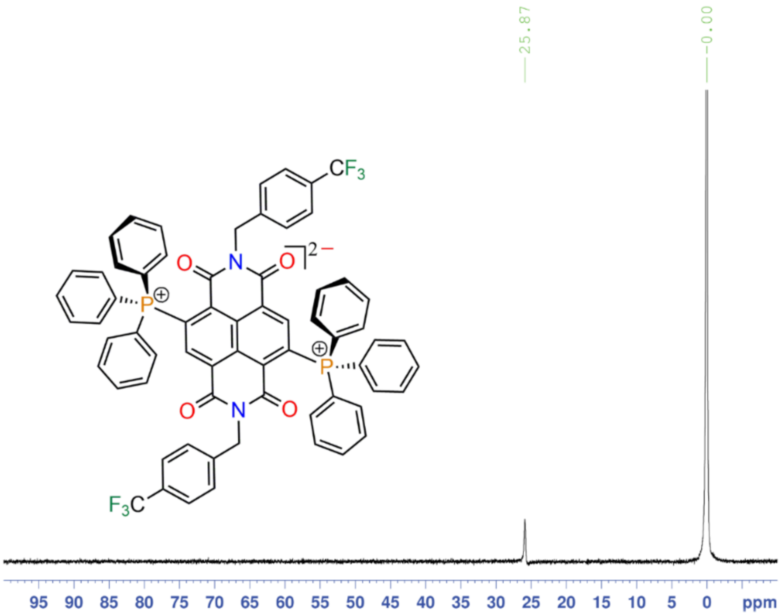

Figure S38: 202 MHz [13]P NMR spectrum of molecule **4** at RT in $CDCl_3$.

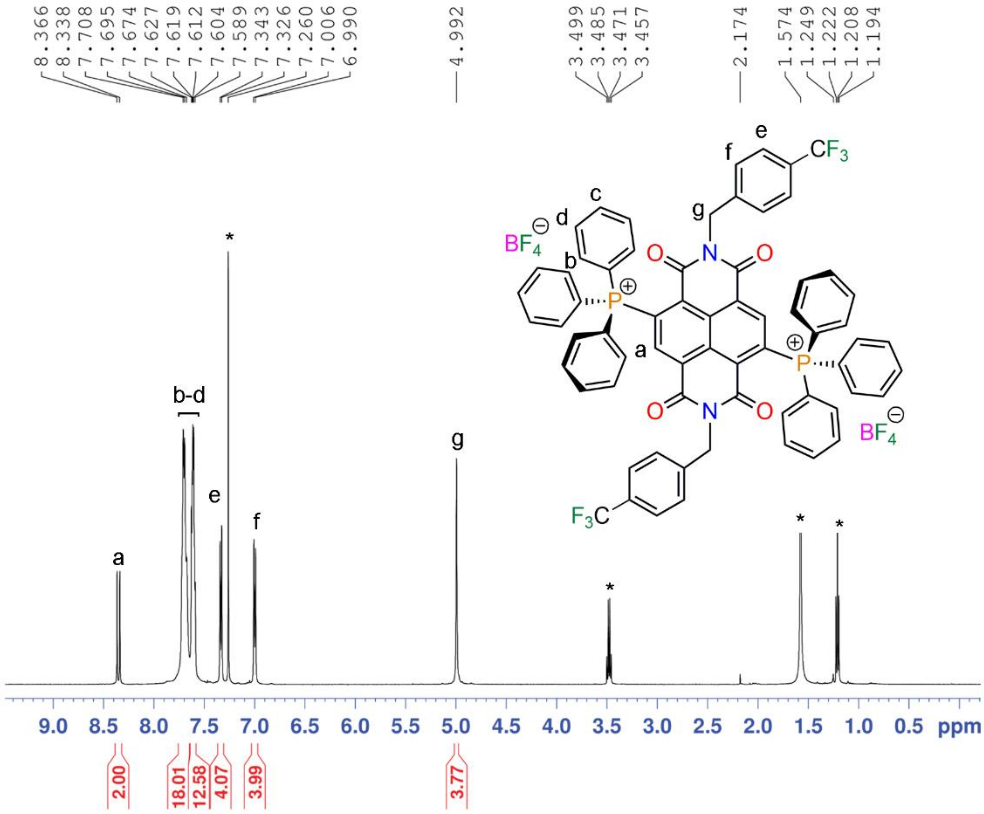

Figure S39: 500 MHz ^{1}H NMR spectrum of molecule $\mathbf{4^{2+}.2BF_4^{2-}}$ at RT in $CDCl_3$.

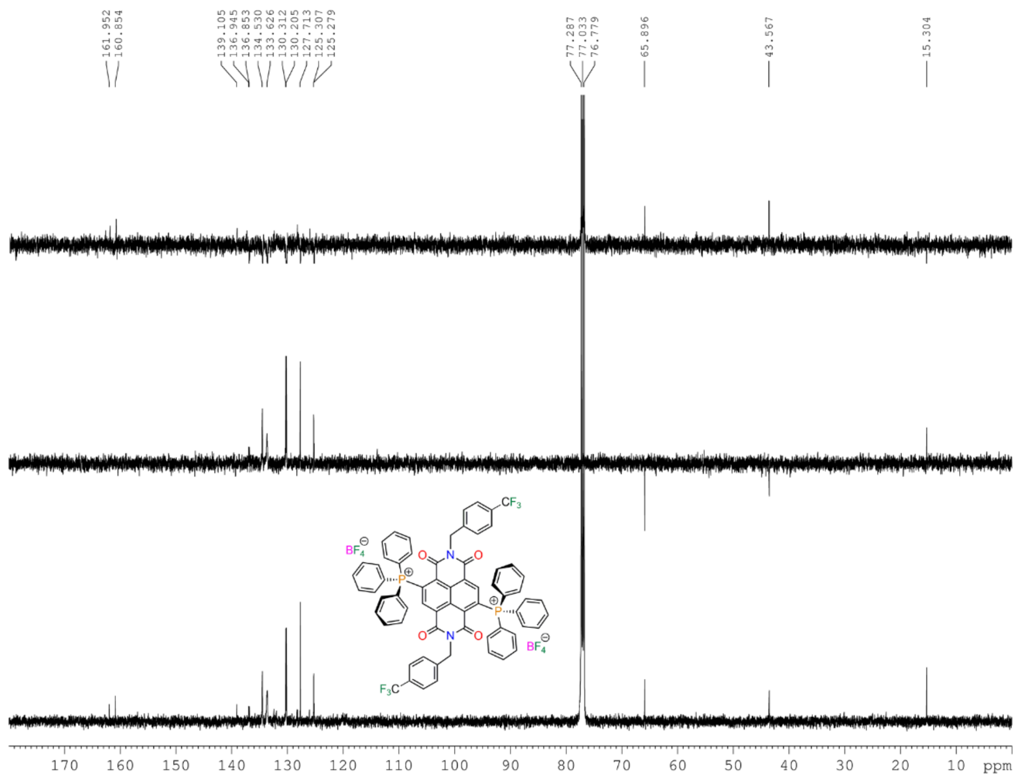

Figure S40: 125 MHz ^{13}C NMR, APT and DEPT-135 spectra of molecule **4.2BF$_4$$^{2-}$** at RT in CDCl$_3$.

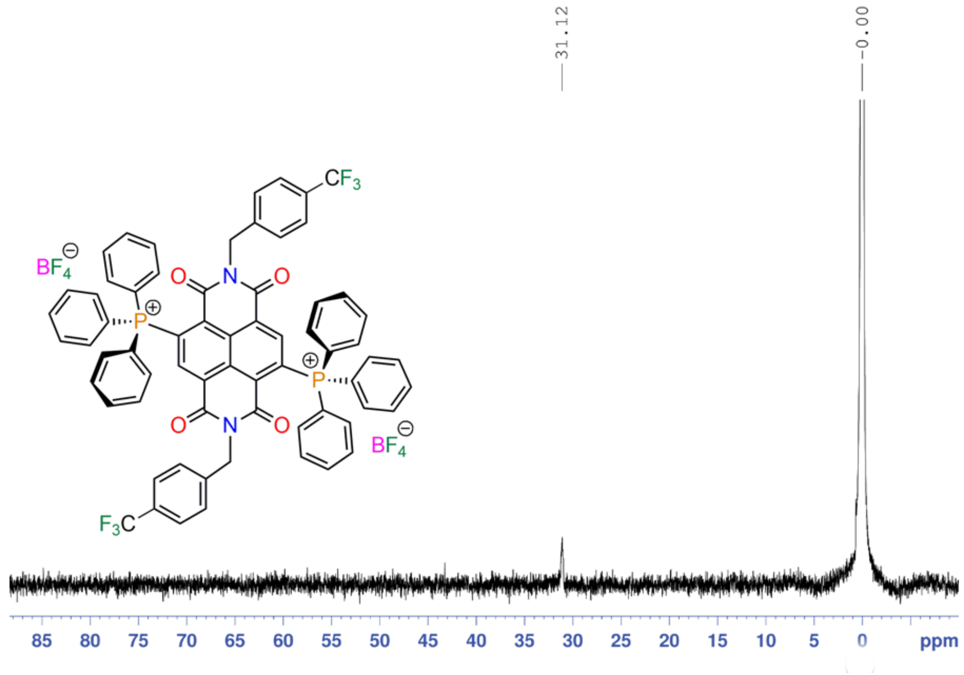

Figure S41: 202 MHz ^{13}P NMR spectrum of molecule **4^{2+}.2BF$_4$$^{2-}$** at RT in CDCl$_3$.

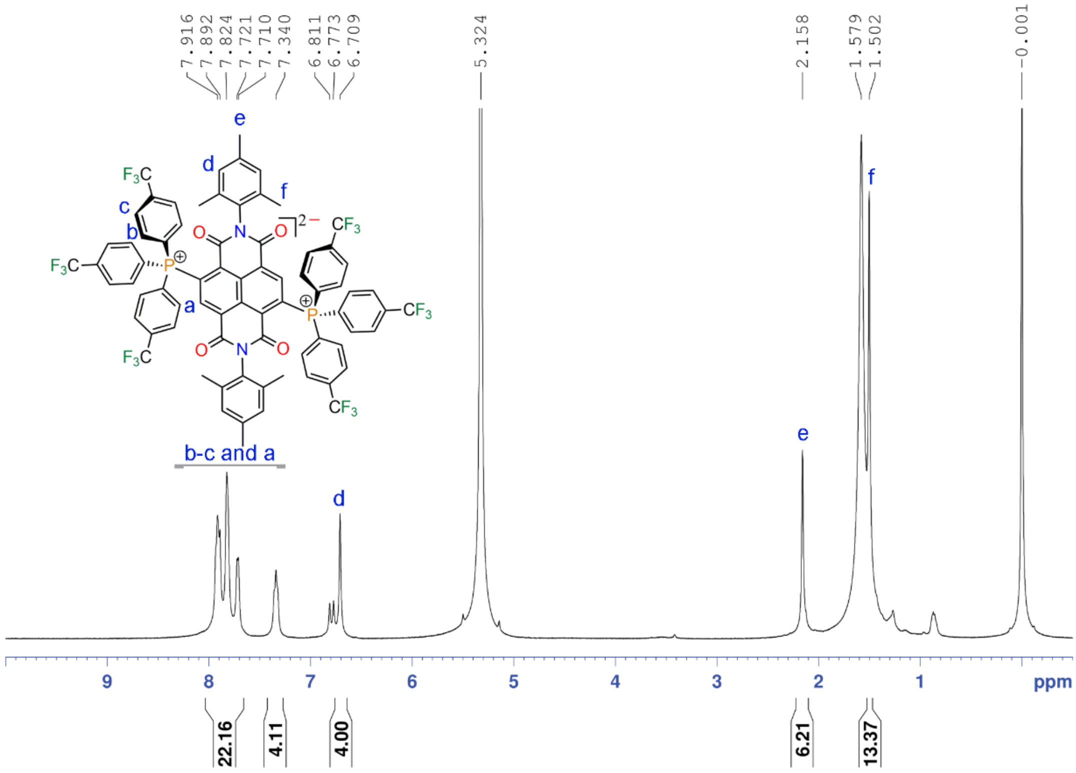

Figure S42: 500 MHz ^{1}H NMR spectrum of molecule **5** at RT in CD_2Cl_2.

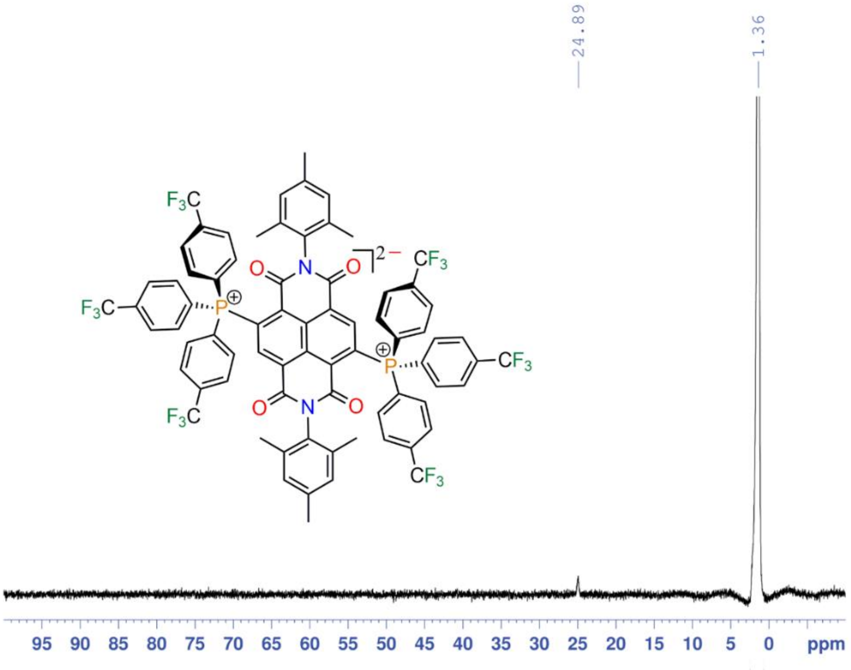

Figure S43: 202 MHz ^{13}P NMR spectrum of molecule **5** at RT in CD_2Cl_2.

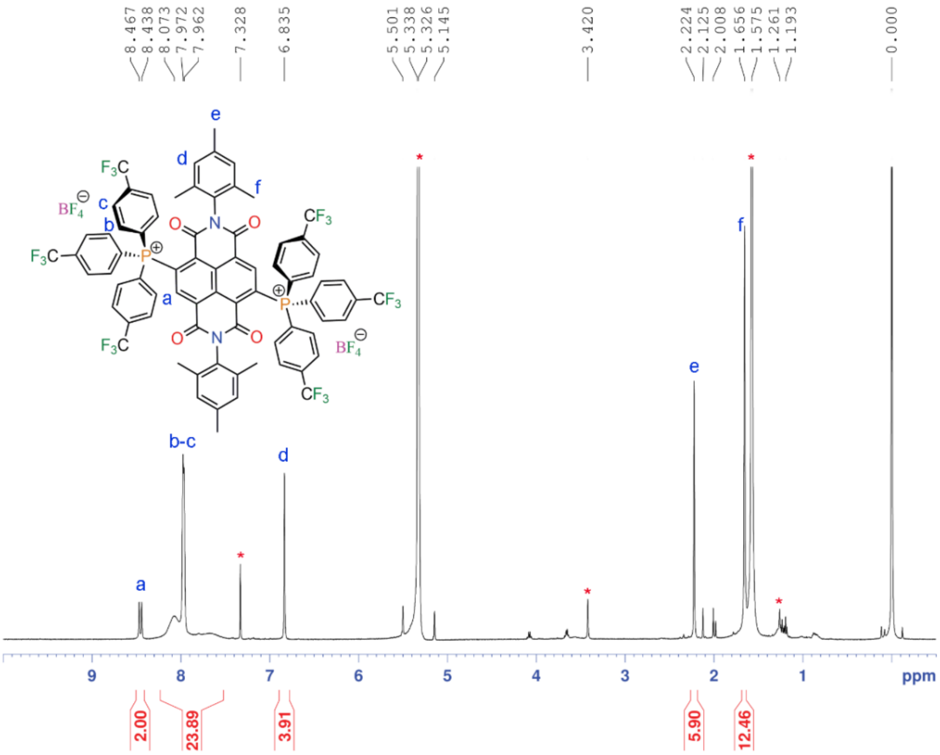

Figure S44: 500 MHz ^{1}H NMR spectrum of molecule **5^{2+}.2BF$_4$$^{2-}$** at RT in CD_2Cl_2.

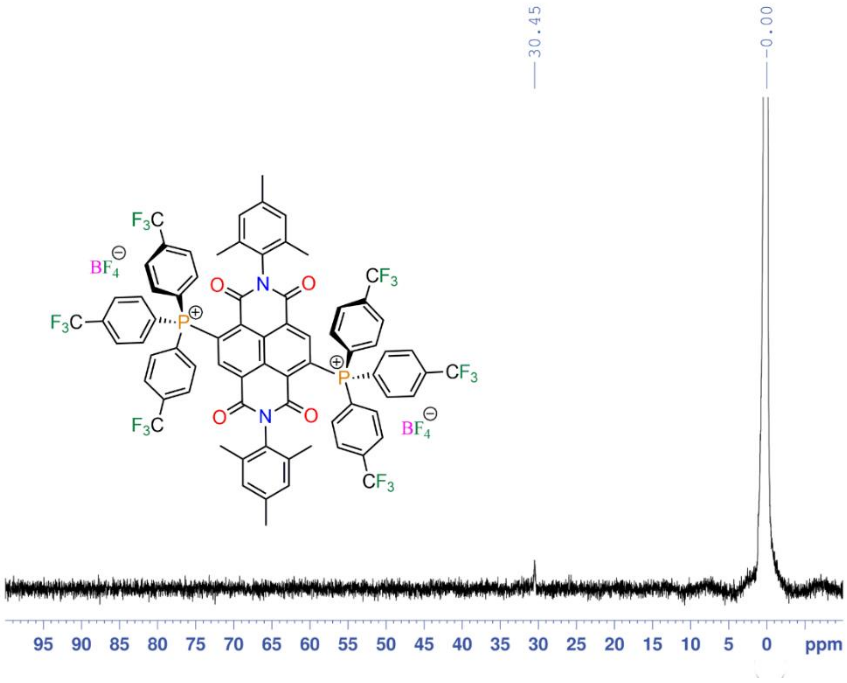

Figure S45: 202 MHz ^{13}P NMR spectrum of molecule **5^{2+}.2BF$_4$$^{2-}$** at RT in CD_2Cl_2.

References:

1. A. D. Becke, *J. Chem. Phys.,* 1993, **98**, 5648.
2. C. Lee, W. Yang and R. G. Parr, *Phys. Rev.* B, 1988, **37**, 785.
3. Gaussian 09, Revision D.01, M. J. Frisch, G. W. Trucks, H. B. Schlegel, G. E. Scuseria, M. A. Rob, J. R. Cheeseman, G. Scalmani, V. Barone, B. Mennucci, G. A. Petersson, H. Nakatsuji, M. Li, X. Caricato, H. P. Hratchian, A. F. Izmaylov, J. Bloino, G. Zheng, J. L. Sonnenberg, M. Hada, M. Ehara, K. Toyota, R. Fukuda, J. Hasegawa, M. Ishida, T. Nakajima, Y. Honda, O. Kitao, H. Nakai, T. Vreven, Jr. J. A. Montgomery, J. E. Peralta, F. Ogliaro, M. Bearpark, J. J. Heyd, E. Brothers, K. N. Kudin, V. N. Staroverov, R. Kobayashi, J. Normand, K. Raghavachari, A. Rendell, J. C. Burant, S. S. Iyengar, J. Tomasi, M. Cossi, N. Rega, J. M. Millam, M. Klene, J. E. Knox, J. B. Cross, V. Bakken, C. Adamo, J. Jaramillo, R. Gomperts, R. E. Startmann, O. Yazyev, A. J. Austin, R. Cammi, C. Pomelli, J. W. Ochterski, R. L. Martin, K. Morokuma, V. G. Zakrzewski, G. A. Voth, P. Salvador, J. J. Dannenberg, S. Dapprich, A. D. Daniels, Ö. Farkas, J. B. Foresman, J. V. Ortiz, J. Cioslowski and D. J. Fox, *Gaussian, Inc., Wallingford CT*, 2009.
4. R. Gershoni-Poranne and A. Stanger, *Chem. Eur. J.,* 2014, **20**, 5673.
5. C. Chuit, R. J. P. Corriu, P. Manforte, C. Reyé, J.-P. Declercq and A. Duborg, *Angew. Chem., Int. Ed. Engl.,* 1993, **32**, 1430.
6. J. Juse´lius, D. Sundholm and J. Gauss, *J. Chem. Phys*., 2004, **121**, 3952; b) H. Fliegl, S. Taubert, O. Lehtonen and D. Sundholm, *Phys. Chem. Chem. Phys*., 2011, **13**, 20500; c) D. Sundholm, H. Fliegl and R. J. F. Berger, Wires *Comput. Mol. Sci.,* 2016, DOI:10.1002/WCMS.1270; d) S. Taubert, D. Sundholm and J. Juse´lius, *J. Chem. Phys*., 2011, **134**, 054123:1–12.
7. R. Ditchfield, Mol. Phys., 1974, 27, 789–807; b) K. Wolinski, J. F. Hinton and P. Pulay, J. Am. Chem. Soc.,1990, 112, 8251–8260.
8. a) Ahrens, James, Geveci, Berk, Law, Charles, ParaView: An End-User Tool for Large Data Visualization, Visualization Handbook, Elsevier, 2005, ISBN-13: 978-0123875822; b) Ayachit, Utkarsh, The ParaView Guide: A Parallel Visualization Application, Kitware, 2015, ISBN 978-1930934306.
9. a) A. E. Read, L. A. Curtiss and F. Weinhold, *Chem. Rev.,* 1988, **88**, 899; b) F. Weinhold and C. R. Landis, Discovering Chemistry with Natural Bond Orbital, *John Wiley & Sons*, 2012.
10. a) R. F. W. Bader, Atoms in Molecules: A Quantum Theory, *Oxford University Press: New York* 1990; b) P. L. A. Popelier, Atoms in Molecules: *An Introduction: Pearson Education, Harlow,* 2000; c) R. J. Gillespie and P. L. A. Popelier, *Chemical bonding and Molecular Geometry; Oxford Universsity Press*: New York, 2001.
11. a) A. D. Becke and K. E. Edgecombe, *J. Chem. Phys.,* 1990, **92**, 5397; b) A. Savin, R. Nesper, S. Wengert and T. F. Fässler, *Angew. Chem. Int. Ed. Engl.,* 1997, **36**, 1809.
12. T. Lu and F. W. Chen, *J. Comput. Chem.,* 2012, **33**, 580.
